# Efficacy and safety of innate and adaptive immunotherapy combined with standard of care in high-grade gliomas: a systematic review and meta-analysis

**DOI:** 10.3389/fimmu.2023.966696

**Published:** 2023-07-06

**Authors:** Baofeng Guo, Shengnan Zhang, Libo Xu, Jicheng Sun, Wai-Lun Chan, Pengfei Zheng, Jinnan Zhang, Ling Zhang

**Affiliations:** ^1^ Department of Plastic Surgery, China-Japan Union Hospital of Jilin University, Changchun, China; ^2^ Department of Pathophysiology, College of Basic Medical Sciences of Jilin University, Changchun, Jilin, China; ^3^ Department of Applied Biology and Chemical Technology, The Hong Kong Polytechnic University, Hong Kong, Hong Kong SAR, China; ^4^ Department of Neurosurgery, China-Japan Union Hospital of Jilin University, Changchun, China

**Keywords:** glioma, glioblastoma, temozolomide, standard of care, radiotherapy, immunotherapy, high-grade, meta-analysis

## Abstract

**Background:**

Malignant glioma is the most common intracranial malignant tumor with the highest mortality. In the era of immunotherapy, it is important to determine what type of immunotherapy provides the best chance of survival.

**Method:**

Here, the efficacy and safety of immunotherapy in high-grade glioma (HGG) were evaluated by systematic review and meta-analysis. The differences between various types of immunotherapy were explored. Retrieved hits were screened for inclusion in 2,317 articles. We extracted the overall survival (OS) and progression-free survival (PFS) hazard ratios (HRs) as two key outcomes for examining the efficacy of immunotherapy. We also analyzed data on the reported corresponding adverse events to assess the safety of immunotherapy. This study was registered with PROSPERO (CRD42019112356).

**Results:**

We included a total of 1,271 patients, of which 524 received a combination of immunotherapy and standard of care (SOC), while 747 received SOC alone. We found that immunotherapy extended the OS (HR = 0.74; 95% confidence interval [CI], 0.56−0.99; *Z* = −2.00, *P* = 0.0458 < 0.05) and PFS (HR = 0.67; 95% CI, 0.45−0.99; *Z* = −1.99, *P* = 0.0466 < 0.05), although certain adverse events occurred (proportion = 0.0773, 95% CI, 0.0589-0.1014). Our data have demonstrated the efficacy of the dendritic cell (DC) vaccine in prolonging the OS (HR = 0.38; 95% CI, 0.21−0.68; Z = −3.23; *P* = 0.0012 < 0.05) of glioma patients. Oncolytic viral therapy (VT) only extended patient survival in a subgroup analysis (HR = 0.60; 95% CI, 0.45−0.80; *Z* = −3.53; *P* = 0.0004 < 0.05). By contrast, immunopotentiation (IP) did not prolong OS (HR = 0.69; 95% CI, 0.50−0.96; *Z* = −2.23; *P* = 0.0256).

**Conclusion:**

Thus, DC vaccination significantly prolonged the OS of HGG patients, however, the efficacy of VT and IP should be explored in further studies. All the therapeutic schemes evaluated were associated with certain side effects.

**Systematic review registration:**

https://www.crd.york.ac.uk/PROSPERO/display_record.php?RecordID=112356.

## 1 Introduction

Gliomas are the most common malignant tumor of the central nervous system (CNS) (1). According to the World Health Organization (WHO) classification of CNS tumors, gliomas can be classified into four grades: Grade 1 and Grade 2 define low-grade glioma (LGG), while Grade 3 and Grade 4 define high-grade gliomas (HGG) (2). The 2021 WHO classification further underscores the role of molecular signatures in stratifying glioma patients, in light of their effects on tumor biology. Thus, the classification incorporates criteria from the 2016 fourth edition, to facilitate the accurate diagnosis, prognosis estimation, and management of the patients with gliomas (1, 3). For example, the presence of a homozygous *CDKN2A/B* deletion results in the WHO Grade 4 classification of the isocitrate dehydrogenase (IDH)-mutant astrocytoma, even in the absence of high-grade histological features (3, 4).

HGGs have a dismal prognosis and are typically considered as incurable. In China, the incidence of HGG is about 5−8/100,000 individuals, and the 5-year mortality rate ranks third among solid tumors, after pancreatic cancer and lung cancer (2). In the U.S., HGG accounts for approximately 80.5% of the 24,560 new cases of malignant primary CNS tumors reported each year (5). Glioblastoma (GBM), as the most common type of WHO Grade 4 glioma, accounts for > 50% of HGGs, with a recurrence rate close to 100%, a 5-year survival rate of < 5%, and a median survival duration of ~ 15−17 months (6, 7). Standard of care (SOC) for HGG usually entails maximal surgical resection, followed by radiotherapy plus chemotherapy, administration of temozolomide (TMZ) as a front-line treatment or the PCV (procarbazine plus lomustine plus vincristine) scheme as an alternative strategy. Sometimes, bevacizumab can also be used as a targeted adjuvant therapy.

Immunotherapy, such as dendritic cell (DC) vaccination, chimeric antigen receptor T (CAR T) cell, immune checkpoint inhibitor (ICI), cytokine therapy, and viral therapy (VT) have gained much research attention and achieved great success in cancer treatment. In recent years, more evidence has shown that HGG patients can benefit from immunotherapy (8, 9). Currently, an immunotherapy using autologous, genetically-modified gamma-delta T cells is being investigated in a clinical trial of GBM (NCT05664243). Phase I studies of avelumab recruited six patients to complete the safety study (NCT02968940); a progression-free survival (PFS) of 4.2 months and an overall survival (OS) of 10.1 months was achieved. A phase II study of nivolumab recruited 26 patients to complete the toxicity study, with a PFS of 4.1 months and an OS of 7.3 months (NCT02550249). A phase II study of durvalumab (NCT02336165) showed that bevacizumab-naïve subjects with GBM who received durvalumab had a longer OS (4.5 months) than bevacizumab-refractory subjects (2 months). Meanwhile, compared with the above trials, DC therapies such as ICT-107(NCT01280552) and DCVax^®^-L were the most effective at improving survival; DCVax^®^-L has recently been approved for a phase III trial (NCT00045968). In addition, microsatellite instability arises in GBM during TMZ treatment, which induces TMZ resistance but promotes the response to ICIs (10). Therefore, immunotherapy may be a promising adjuvant for alleviating resistance to chemotherapy in HGG.

The immunotherapeutic interventions discussed in the current systematic review are categorized as follows:

1. Boosting adaptive immunity: DC vaccination (11–13) and oncolytic VT (AdvHSV-tk and PVSRIPO) (14–19).
2. Boosting innate immunity: Immunopotentiators (IP) such as transforming growth factor (TGF)-β2 anti-sense oligonucleotide (ODN) and Cpg-ODN (20, 21).

To evaluate the efficacy and safety of the combination of immunotherapy and SOC vs. SOC alone, this meta-analysis utilized patient survival data from published papers. We hope that our findings will help inform clinicians and scientists about the types of immunotherapy of most benefit to HGG patients.

## 2 Materials and methods

### 2.1 Search strategy and selection criteria

An electronic search was performed by two authors (B.F. Guo and J.C. Sun) using single terms and phrases through the four databases, Cochrane Library, Embase, PubMed, and Web of Science, for relevant articles published up to January 1^st^, 2020. Search terms included “high grade glioma”, “astrocytoma”, “glioblastoma”, “immunity”, “immunotherapy”, “humans” and “randomized”. An English language restriction was included. Clinical trials are registered on the website http://ClinicalTrials.gov were also explored.

The following inclusion criteria were adopted: (1) phase II-III clinical trials including at least two arms and its therapeutic intervention restricted to immunotherapy; (2) studies including adult patients (age ≥ 18) with HGG according to standardized diagnostic criteria; (3) studies comparing immunotherapy with SOC treatment. Studies were included when they meet all inclusion criteria. While studies were excluded when they meet any exclusion criteria including (1) studies lacking relevant outcome data; (2) trials without the SOC control arms; (3) phase I trials without NCT numbers; (4) phase II single-arm trials; (5) animal trials or cell assay; (6) abstracts and presentations from all major conference.

### 2.2 Data extraction and quality assessment

Two investigators (L.B. Xu and S.N. Zhang) extracted relevant information from the included articles. The hazard ratio (HR) has been described as a more suitable measure for analyzing time-to-event outcomes than the odds ratio or relative risk, and thus, the HR data were extracted (22, 23). When reports of HR and 95% confidence interval (CI) were not available, the estimated value was derived directly from Kaplan-Meier curves according to the methodology described by Jayne F Tierney (23). Dot plots of the graphical data were extracted *via* Engauge Digitizer 4.1 software (http://digitizer.sourceforge.net/). Most adverse events (AEs) were collected according to NCI-CTC 2.0/3.0.

### 2.3 Statistical analysis

Our statistical analyses were performed by R (version 3.6.1 for Windows; https://www.r-project.org/). The specific protocol for operation has been previously published (24). The main endpoints were OS, PFS, and AEs. The HR and 95% CIs were calculated for OS and PFS. The risk ratio (RR) and 95% CIs was calculated for part of AE, while other AEs with single-arm statistic materials were calculated by *Proportion* and 95% CIs. A random-effect model was used when the studies present significant heterogeneity. A fixed-effect model was used for those studies without significant heterogeneity. Heterogeneity across trials was assessed with the *I^2^* test, and *I^2^* > 50% and *P*<0.05 suggested that there was significant heterogeneity. Publication bias was examined by funnel plots. The specific information about types of immunotherapies, lesions, allocation methods, and so on was analyzed and discussed *via* a detailed subgroup analysis. Sensitivity analysis was performed to explore the impact of each individual study by removing one study at a time. Publication bias was examined by funnel plots.

## 3 Results

### 3.1 Trial selection

**Figure 1:**
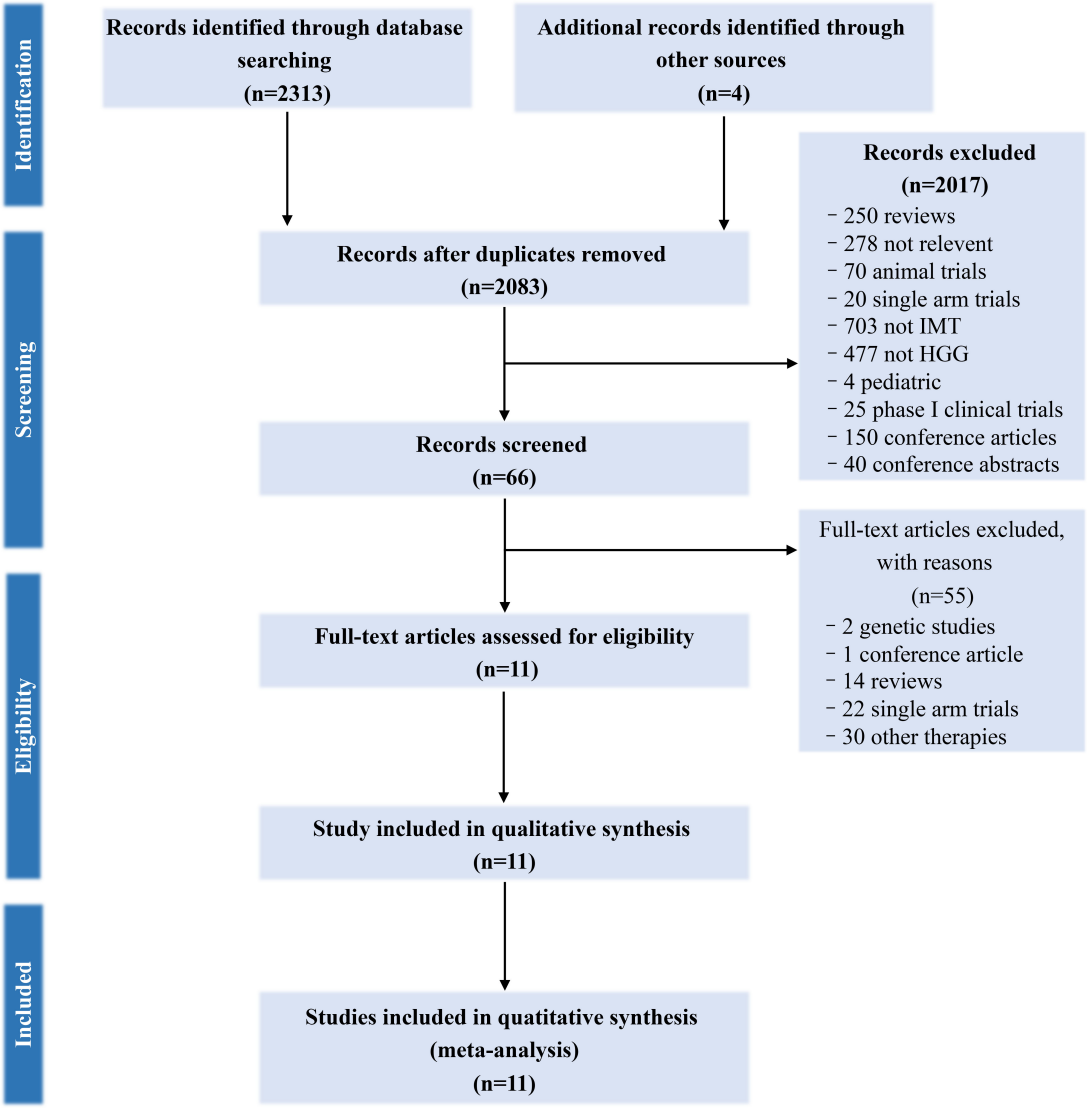
PRISMA (Preferred Reporting Items for Systematic Reviews and Meta-Analyses) flow-chart of search strategy.

Overall, 2,315 citations were identified by the researchers, and 66 potentially eligible articles were retrieved in full text. 55 of them were excluded but four additional studies were included from another similar meta-analysis, resulting in 11 studies describing the efficacy of immunotherapy between 2004 and 2018. The literature screening process was shown in **Figure 1**. The Cochrane risk assessment form was used to evaluate the quality of the research. Nine of the 11 studies described the use of randomized control, the random sequence generation method was a random number table method, and two of them used the intent-to-treat (ITT) patients’ baseline data to match similar historical patients’, so they were evaluated as high-risk. Two of the 11 studies used the double-blind method, three of which were clearly described as open experiments, and the rest of the undescribed evaluations were unclear. Two studies describe the blind method of participants and none of the others does. Four studies had missing data for follow-up, so the incomplete report was evaluated as high risk. Two articles with incomplete reports were evaluated as high risk, and the rest were unclear. The quality of the included studies was evaluated as grade C. The evaluation details are shown in **Supplementary Figure 1** and **Supplementary Figure 2**.

### 3.2 Main characteristics of the studies

We classified these studies into groups according to the classification of immunotherapy types, such as oncolytic VT (AdvHSV-tk + GCV/PVSRIPO), DC vaccination (autologous DC/mRNA-transfected DC/CD133 specific DC), and IPs (trabedersen/CpG oligonucleotide). A total of 524 participants were subjected to immunotherapy, and 747 participants were provided with SOC (a total of 1,271 participants).

A total of 914 participants from VT studies (393 patients in the experimental arm and 521 patients in the control arm) were included. Three of the studies containing TMZ in the SOC regimen also reported PFS. The details can be found in **Table 1**. There were three studies on DC therapy. All studies used TMZ in the SOC regimen. 90 participants underwent DC therapy (43 patients in the experimental arm and 47 patients in the control arm) (**Table 2**). IP was used in two studies that applied TMZ in the SOC regimen (88 patients in the experimental arm and 179 patients in the control arm) (**Table 3**).

**Table 1:** Main Characteristics of studies that use viral therapy (VT) for the treatment of HGGs.

Study name	NCT number	Pha-se	Patients (E/C)	Age (E)	Age (C)	Intervention	Control	KPS score	Region	Patients Characte-ristics	Follow-up Time	EM OS	CM OS	EM PFS	CM PFS	SOC
Rainov 2000	N/A	III	121/116	60	58	RVHSV-tk+GCV +SG+RT	SG+RT	≥70	Europe	Newly diagnosed GBM	21.6	12.2	11.8			VT+ SOC (SG+RT)
Immonen 2004	N/A	II	17/19	52	56.5	AdvHSV-tk+GCV +SG+RT	SG+RT	≥70	Europe	PR/REC HGG	56	15.5	9.4			VT+ SOC (SG+RT)
Ji 2015	N/A	II	22/22	49	54	AdvHSV-tk +GCV+SG	SG+RT+ chemo	N/A	China	Recurrent HGG	60	10.6	3.3	6.9	2.0	VT+ SOC (SG+RT+ chemo)
Westphal 2013	2004-000464-28^a^	III	124/126	58	57	AdvHSV-tk+GCV +SG+RT+TMZ (48.7%)	SG+RT+ TMZ (65%)	≥70	Europe	Newly diagnosed GBM	46	16.6	15.1			VT+ SOC (SG+RT +partly chemo)
Wheeler 2016	NCT00589875	II	48/134	57	60	AdvHSV-tk+GCV +SG+RT+TMZ	SG+RT+ TMZ	≥70	USA	Newly diagnosed HGG	60	17.1	13.5	8.1	6.5	VT+ SOC (SG+RT+ chemo)
Desjardins 2018	NCT01491893	II	61/104	55	55	PVSRIPO+Bev+SG+chemotherapy+RT	Bev+ SG+ chemotherapy +RT	≥70	USA	Recurrent GBM	27.6	11.7	10.5			VT+ SOC (SG+RT+ chemo)

E, experiment group; C, control group; Age, median age; MOS, median survival; MPFS, median progression-free survival; N/A, not available; SG, surgery; RT, radiotherapy.

**Table 2:** Main Characteristics of studies that use DC therapy for the treatment of HGGs.

Study name	NCT number	Pha-se	Patient (E/C)	Age (E)	Age (C)	Intervention	Control	KPS score	Region	Patients Characte-ristics	Follow-up Time	EM OS	CM OS	EM PFS	CMPFS	SOC
Cho 2012	N/A	II	16/18	58	58.5	DCV+ SG+RT+TMZ	SG+RT+TMZ	>70	China	Newly diagnosed GBM	56	31.9	15.0	8.5	8.0	DC+ SOC (SG+RT+ chemo)
Vik-mo 2013	NCT00846456	N/A	6/7	57	56	DCV+ SG+RT+TMZ	SG+RT+TMZ	N/A	Europe	Newly diagnosed GBM	33	25.3	19.5	23.1	7.9	DC+ SOC (SG+RT+ chemo)
Yao 2018	NCT01567202	II	21/22	48	50	DCV+ SG+RT+TMZ	SG+RT+TMZ	≥60	China	PR/REC GBM	14	13.7	10.7	7.7	6.9	DC+ SOC (SG+RT+ chemo)

E, experiment group; C, control group; Age, median age; MOS, median survival; MPFS, median progression-free survival; N/A, not available; SG, surgery; RT, radiotherapy.

**Table 3:** Main Characteristics of studies that use immunopotentiator therapy for the treatment of HGGs.

Study name	NCT number	Pha-se	Patient (E/C)	Age (E)	Age (C)	Interven -tion	Con -trol	KPS score	Region	Patients Characteristics	Follow-up Time	EMOS	CMOS	EM PFS	CM PFS	SOC
Ursu 2017	NCT00190424	II	39/42	62	59	CpG-ODN+ SG+ RT+TMZ	SG+ RT+ TMZ	≥60	Europe	Recurrent GBM	60.0	15.9	16.8	9.0	8.5	IP+ SOC ( SG+RT+ chemo)
Bogdahn 2011	N/A	IIb	40/45	51.5	48	TGF-β2 inhibitor +SG+RT+ TMZ	SG+ PCV+ TMZ	≥70	Europe	Recurrent GBM/AA	70.0	39.1	21.7			IP+ SOC ( SG+RT+ chemo)

E, experiment group; C, control group; Age, median age; MOS, median survival; MPFS, median progression-free survival; N/A, not available; SG, surgery; RT, radiotherapy.

### 3.3 Efficacy and safety analysis of immunotherapy

#### 3.3.1 Overall survival

OS data was extracted from 11 studies with 1,271 participants. However, substantial heterogeneity was found, which shows the variability among the OS data (*τ^2 =^* 0.1315; *I^2 =^* 65 > 50%; *P* < 0.05). Thus, we employed a random-effect model to assess the efficacy of extending OS. It showed that immunotherapy decreased the risk of death by 26% compared with the SOC (HR = 0.74; 95% CI, 0.56−0.99; *P* = 0.0458; **Supplementary Figure 3**). Sensitivity analysis was performed to assess how each study influenced efficacy estimates (**Supplementary Figure 4**), and it showed our result was stable. In the funnel plot, it was found that there was a substantial publication bias, so the trim and fill method was used to adjust publication bias with a new effect estimate by complementing four studies. Unexpectedly, it showed that compared with SOC, immunotherapy did not improve the OS of patients (HR = 0.98; 95% CI, 0.72−1.34; *Z* = −2.00; *P* = 0.90 > 0.05) (**Supplementary Figure 5**).

##### 3.3.1.1 Subgroup analysis by therapeutic schemes

**Figure 2:**
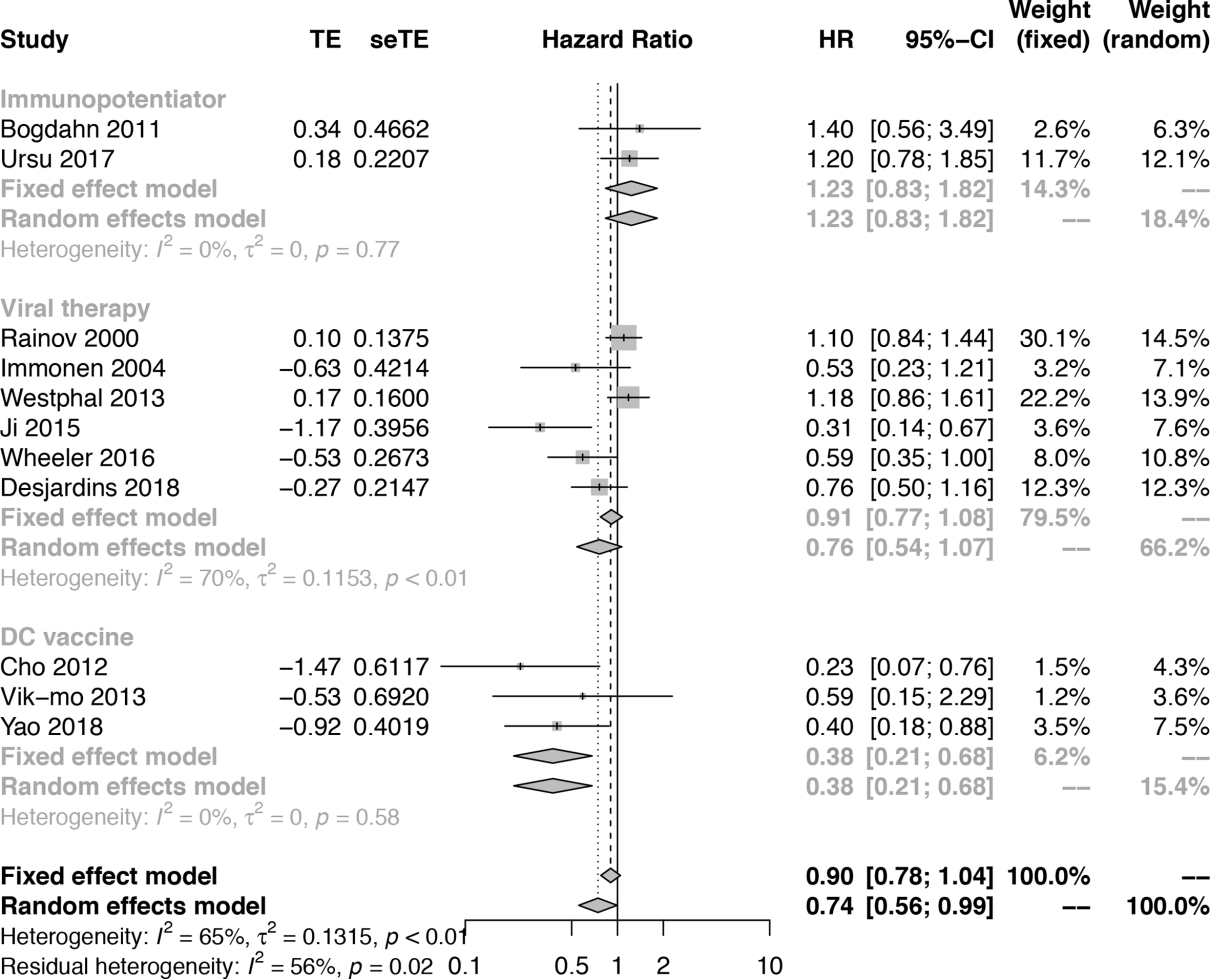
Subgroup analysis of the combination of immunotherapy and standard of care compared with standard of care according to therapeutic scheme.

To further understand the efficacy of immunotherapy for HGGs, subgroup analysis was performed by therapeutic schemes. It was obvious that VT and IP did not prolong the OS [(HR = 0.76; 95% CI, 0.54−1.07; *P* > 0.05); (HR = 1.23; 95% CI, 0.83−1.82; *P* > 0.05)], while DC therapies prolonged OS significantly (HR = 0.38; 95% CI, 0.21−0.68; *P* = 0.0012 < 0.05] (**Figure 2**). The results were basically consistent with the previous reports (**Supplementary Table 1**).

##### 3.3.1.2 Subgroup analysis by lesion type

To assess the efficacy of immunotherapy for different types of HGGs, subgroups analysis according to lesion type (recurrent/primary and type of glioma) was performed but showed no significant difference between subgroups, which might attribute to the limited number of studies (*Q* = 11.08; *P* = 0.085 > 0.05) (**Supplementary Figure 6**).

##### 3.3.1.3 Subgroup analysis by implementary plan of clinical trials

To explore the relationship between the implementary plan of clinical trials and efficacy, subgroup analysis was performed. It showed that there was no significant difference between the open-label trials and double-blind trials (*Q* = 1.22; *P* = 0.2703 > 0.05) (**Supplementary Figure 7**). Besides, it was noted that the efficacy of observational historical matched studies which match historical patients’ baseline information to mimic randomization was better than randomized controlled trial (RCT) studies which also implied the difference between RCT and historical matched studies (*Q* = 0.18; *P* = 0.673 > 0.05) (**Supplementary Figure 8**).

##### 3.3.1.4 Subgroup analysis by recruiting area of clinical trials

According to the recruiting area (multi-center, Europe, China, and the United States), there was a significant difference between the subgroups, and the difference was statistically significant (*Q* = 23.67, *P* < 0.0001) (**Supplementary Figure 9**). The results showed that studies carried out in China and the United States had a better effect on prolonging OS [(HR = 0.33; 95% CI, 0.20−0.54; *P* < 0.05); (HR = 0.69; 95% CI, 0.50−0.96; *P* < 0.05)].

##### 3.3.1.5 Subgroup analysis by the intervention of SOC

As the intervention of SOC is also crucial for the efficacy of immunotherapy, the subgroups were divided by the intervention of SOC (TMZ/No TMZ). It showed that there was a significant difference between subgroups (*Q* = 4.33; *P* = 0.0374 < 0.05). It suggested that TMZ synergized with immunotherapy (HR=0.63, 95% CI, 0.43−0.94; *P* = 0.01 < 0.05), (**Supplementary Figure 10**).

Overall, the influence factors were examined and summarized according to the defined characteristics of interventions, clinical trials, lesions, and study design (**Table 4**).

**Table 4:** Subgroup analysis of IMT and death incidence for aach variables.

Variable	No. of Studies	No. of Participants		OS, HR(95%CI)	I^2^ value(%)	*p* value^c^
		IMT^a^	SOC^b^			
Therapy type						
DC vaccine	3	43	47	0.38 [0.21;0.68]	0	0.004**
Immunopotentiator	2	88	179	1.23 [0.83;1.82]	0	
Viral therapy	6	393	521	0.76 [0.54;1.07]	70	
Lesion Type						
Newly diagnosed GBM	3	143	141	0.60 [0.22;1.64]	71	0.085
Primary and recurrent GBM	1	21	22	0.53 [0.23;1.21]	/	
Newly diagnosed HGG	2	172	260	0.86 [0.44;1.70]	80	
Recurrent HGG	1	22	22	0.31 [0.14;0.67]	/	
Recurrent GBM	2	109	238	0.95 [0.61;1.49]	55	
Primary and recurrent HGG	1	17	19	0.40 [0.18;0.88]	/	
Recurrent AA	1	40	45	1.40 [0.56;3.49]	/	
Label type						
Open-label	8	331	465	0.66 [0.46; 0.96]	63	0.270
Double Blind	3	193	282	0.93 [0.57; 1.54]	70	
Study Design						
Randomized	8	409	502	0.76 [0.52; 1.10]	72	0.673
Historical control	3	115	245	0.68 [0.50; 0.94]	0	
Recruiting area						
Multi-center	2	245	242	1.13 [0.92; 1.39]	0	0.001*
Europe	4	111	205	0.96 [0.62; 1.50]	26	
China	3	59	62	0.33 [0.20; 0.54]	0	
USA	2	109	238	0.69 [0.50; 0.96]	0	
SOC Type						
TMZ	8	262	486	0.63 [0.43; 0.94]	56.8	0.037*
Not TMZ	3	262	261	1.05 [0.80; 1.39]	58.1	

^a^IMT, Immunotherapy combined with SOC; ^b^SOC, Standard o Care; ^c^p value for subgroup differences (random effects model was applied in first two variables; fixed effects model was applied in other variables); *p < 0.05; **p < 0.01.

#### 3.3.2 Progression-free survival

As six studies involving a total of 397 participants reported PFS, a random-effect model was used to assess the efficacy of immunotherapy versus SOC according to the HR of PFS. Based on the meta-analysis (**Supplementary Figure 11**) and sensitivity analysis (**Supplementary Figure 12**), the combination of immunotherapy and SOC significantly improved the PFS (HR = 0.67; 95% CI, 0.45-0.99; *Z* = −1.99; *P* = 0.0466 < 0.05). The funnel plot was performed to evaluate the publication bias and the trim and fill method was used. A new combined effect value was obtained by supplementing two studies. The results showed that compared with SOC, immunotherapy did not improve the PFS (HR = 0.83; 95% CI, 0.54−1.28; *Z* = −0.84; *P* = 0.399 > 0.05] (**Supplementary Figure 13**).

##### 3.3.2.1 Subgroup analysis by therapeutic schemes

We wondered if some variables influenced the efficacy of PFS like OS as mentioned before. According to the type of immunotherapy (IP, VT, DC), there was no significant difference between the subgroups (*Q* = 4.84; *P* = 0.089 > 0.05) (**Supplementary Figure 14**).

##### 3.3.2.2 Subgroup analysis by lesion type

Besides, according to lesion type (recurrent GBM, primary and recurrent GBM, newly diagnosed GBM, recurrent HGG, HGG), the results also showed that there was no significant difference between the subgroups (*Q* = 9.46; *P* = 0.051 > 0.05) (**Supplementary Figure 15**).

#### 3.3.3 Adverse events of immunotherapy

A total of eight studies reported on the occurrence of AEs of immunotherapy combined with SOC. The toxic and side effects of immunotherapy combined with conventional treatment were evaluated through a single-arm meta-analysis first. The sample size was 397. After the heterogeneity test (*τ^2 ^= *1.3055; *I^2 =^* 95.5% > 50%; *P* = 0 < 0.05), random-effect model was adopted. It showed immunotherapy combined with SOC had a risk of AEs (proportion = 0.0773; 95% CI, 0.0589−0.1014) (**Supplementary Figure 16**). A total of five studies reported the occurrence of AE in both experiment and control arms so the RR value was used to evaluate the toxicity and side effects of immunotherapy. The sample size was 709. After the heterogeneity test (*τ^2 ^= *1.3055; *I^2 ^= *78.8% > 50%; *P* < 0.0001), a random-effect model was adopted. Meta-analysis results showed that compared with SOC, the risk of AE of immunotherapy combined with SOC increased by 67.37% (RR = 1.67; 95% CI, 1.28−2.19; *Z* = 3.76; *P* = 0.0002 < 0.05), suggesting that immunotherapy had certain toxicity and side effects (**Supplementary Figure 17**).

### 3.4 Efficacy analysis of VT, DC therapy and IP

#### 3.4.1 The efficacy of VT

The HR of OS was used to compare the efficacy of VT combined with SOC and SOC alone. A total of six studies, with a sample size of 913, were tested for meta-analysis (*τ^2 ^= *0.115; *I^2^ = *70.5% > 50%; *P* = 0.0046 < 0.05). It showed that compared with SOC, the combination of VT and SOC had a trend to prolong the OS of patients, but the difference was not statistically significant (HR = 0.76; 95% CI, 0.54−1.07; *Z* = −1.58; *P* = 0.113 > 0.05) (**Supplementary Figure 18**). Sensitivity analysis proved our results were stable (**Supplementary Figure 19**). The funnel plot showed asymmetry so the trim and fill method was used to further evaluate the publication bias. A new combined effect estimate was obtained by supplementing three studies. The results showed that compared with SOC, the combination of VT and SOC did not improve the OS of patients with HGG (HR = 1.03; 95% CI, 0.72−1.48; *Z* = −0.17; *P* = 0.869 > 0.05) (**Supplementary Figure 20**).

The HR of PFS was used to further evaluate the efficacy of VT combined with SOC. There were only two studies with a sample size of 226 cases. After heterogeneity test (*τ^2 =^* 2.12; *I^2 =^* 77.7% > 50%; *P* = 0.0344 < 0.05), random-effect model was used for meta-analysis. The results showed that compared with SOC, the combination of VT and SOC did not prolong the PFS (HR = 0.52; 95% CI, 0.22−1.22; *Z* = −1.50; *P* = 0.1343 > 0.05) (**Supplementary Figure 21**). The progression-free survival HR value was also used for sensitivity analysis which was consistent with the previous result (**Supplementary Figure 22**).

**Figure 3:**
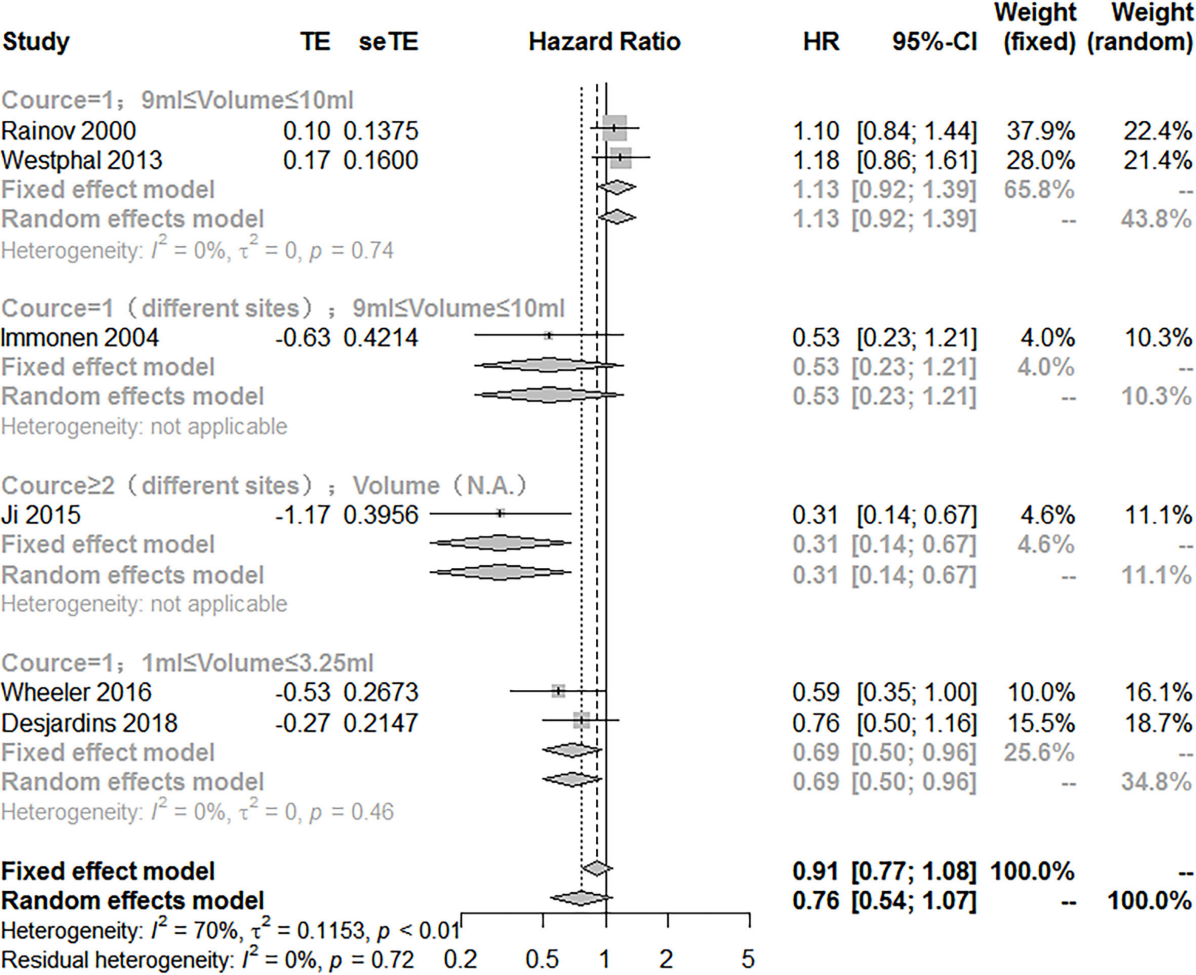
Subgroup analysis of the combination of viral therapy and standard of care compared with standard of care according to therapeutic scheme.

To explore the potency of VT, subgroup analysis according to the injection method of VT (treatment course, injection volume, multipoint injections) showed that there are significant differences between the subgroups (*Q* = 16.27; *P*<0.05). The treatment of VT with more than two courses of treatment and multipoint injections combined with SOC manifested a better effect on improving the OS (HR = 0.31; 95% CI, 0.14−0.67). However, the use of 9~10 ml injection volume of VT and multipoint injections did not exhibit the treatment effect on prolonging the OS [(HR = 0.53; 95% CI, 0.23−1.21); (HR = 1.13; 95% CI, 0.92−1.39)]. Noteworthily, VT with an injection volume of 1~3.5 ml significantly prolonged the OS compared with VT with an injection volume of 9~10 ml [(HR = 0.69; 95% CI, 0.50−0.96); (HR = 1.13; 95% CI, 0.92−1.39)] (**Figure 3**).

**Figure 4:**
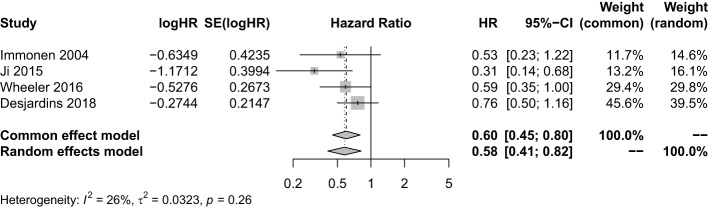
Analysis of the OS of the combination of multiple courses of treatment/multi-point injection/small injection volume viral therapy and standard of care compared with standard of care.

We pooled all trials that employed the injection method including multiple courses of treatment, multipoint injection, and small injection volume of VT. There were four studies with a sample size of 427. It showed that VT with optimized injection methods combined with SOC significantly prolonged the OS (HR = 0.60; 95% CI, 0.45−0.80; *Z* = −3.53; *P* = 0.0004 < 0.05) (**Figure 4**). The sensitivity analysis showed our result was stable. The combined effect size was consistent with the previous results (**Supplementary Figure 23**). However, the funnel plot was asymmetric, suggesting there was a publication bias. Thus, the trim and fill method was used to evaluate the publication bias, and a new combined effect value was obtained by supplementing two studies. The results showed VT with optimized injection methods combined with SOC prolong the OS of HGG patients (HR = 0.68; 95% CI, 0.47−0.99; *Z* = −2.89; *P* = 0.0039 < 0.05) (**Supplementary Figure 24**).

The HR value of PFS was also used to evaluate the efficacy of VT with optimized injection methods combined with SOC. There were two studies with a sample size of 226 cases. It showed that VT with optimized injection methods combined with SOC did not improve the PFS (HR = 0.52; 95% CI, 0.22−1.22; Z = −1.50; *P* = 0.1343 > 0.05) (**Supplementary Figure 25**). Sensitivity analysis showed the combined effect estimate was consistent with the previous results (HR = 0.52; 95% CI, 0.22−1.22; *Z* = −1.50; *P* = 0.1343 > 0.05) (**Supplementary Figure 26**).

#### 3.4.2 The efficacy of DC therapy

The HR of OS was used to evaluate the efficacy of DC therapy combined with SOC. There were three studies with a sample size of 90. It showed DC therapy combined with SOC significantly improved the OS of patients with HGGs (HR = 0.38; 95% CI, 0.21−0.68; *Z* = −3.23; *P* = 0.0012 < 0.05) (**Supplementary Figure 27**). Sensitivity analysis proved our results were stable (**Supplementary Figure 28**). Funnel plot was symmetric. Notwithstanding, the trim and fill method was still used to evaluate the publication bias, and a new combined effect value was obtained by supplementing two studies. It showed that compared with SOC, the combination of DC therapy and SOC improved the OS of patients with HGGs (HR = 0.38; 95% CI, 0.21−0.68; *Z* = −3.23; *P* = 0.0012 < 0.05) (**Supplementary Figure 29**).

The HR of PFS was also used to evaluate the efficacy of DC therapy combined with SOC. There were three studies with a sample size of 90. It showed that compared with SOC, the combination of DC therapy and SOC did not improve the PFS (HR = 0.60; 95% CI, 0.35−1.03; *Z* = −1.84; *P* = 0.066 > 0.05) (**Supplementary Figure 30**). Sensitivity analysis showed the combined effect estimate was consistent with the previous results (**Supplementary Figure 31**).

#### 3.4.3 The efficacy of IP

**Figure 5:**
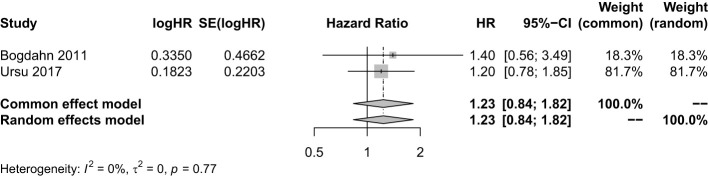
Analysis of the OS of the combination of immunopotentiators and standard of care compared with standard of care.

The HR value of OS was used to compare the efficacy of IP combined with SOC and SOC alone. There were two studies with a sample size of 166. It showed that compared with SOC, the combination of IP and SOC did not improve the OS of patients with HGGs (HR = 1.23; 95% CI, 0.83−1.82; *Z* = 1.05; *P* = 0.2918 > 0.05) (**Figure 5**). Sensitivity analysis showed our result was stable. The combined effect estimate was consistent with the previous results (**Supplementary Figure 32**). The funnel plot was asymmetry so the trim and fill method was still used to evaluate the publication bias, and the results showed that compared with SOC, the combination of IP and SOC did not prolong the OS of patients with HGGs (HR = 1.23; 95% CI, 0.83−1.82; *Z* = 1.05; *P* = 0.2918 > 0.05) (**Supplementary Figure 33**).

### 3.5 Adverse events of different type of immunotherapy

#### 3.5.1 The safety of oncolytic VT

According to the type of immunotherapy, the toxic and side effects of VT combined with SOC were evaluated through a single-arm meta-analysis first. A total of three studies (270 participants) reported the occurrence of AE of VT combined with SOC. After the heterogeneity test (*τ^2 ^= *1.0463; *I^2 ^= *82% > 50%; *P* < 0.05), a random-effect model was adopted. The meta-analysis results show that there was a risk (3%) of AE with the combination of VT and SOC (proportion = 0.03; 95% CI, 0.02−0.05) (**Supplementary Figure 34**), suggesting that the combination of VT and SOC had moderate toxicity and side effects. It was noteworthy that compared with the occurrence of AE with the combination of immunotherapy and SOC (proportion = 0.0773; 95% CI, 0.0589−0.1014), the incidence of AE with the combination of VT and SOC was lower. A total of three studies reported the occurrence of AE in double arms. It showed that compared with SOC, the risk ratio of AE with VT combined with SOC increased by 45.33% (RR = 1.45; 95% CI, 1.18−1.79; *Z* = 3.50; *P* = 0.0005 < 0.05), suggesting that compared with SOC, the combination of VT and SOC still had certain side effects (**Supplementary Figure 35**).

#### 3.5.2 The safety of DC therapy

Next, the toxicity and side effects of DC therapy combined with SOC were evaluated through a single-arm meta-analysis. The sample size was 47. After heterogeneity testing (*τ^2 ^= *0.9188; *I^2 ^= *83.4% > 50%; *P* < 0.05), random-effect model was used. Single-arm meta-analysis results showed that there was a risk (18%) of AE in the combination of DC therapy and SOC [proportion = 0.1800, 95% CI 0.0959−0.3379] (**Supplementary Figure 36**). It should be noted that with the sample size increasing, the probability of AE can be increased proportionally. Compared with the incidence of AE in immunotherapy and SOC (proportion = 0.0773; 95% CI, 0.0589−0.1014], the incidence of AE in DC therapy and SOC was higher.

#### 3.5.3 The safety of IP

Finally, the toxicity and side effects of IP combined with SOC were evaluated through a single-arm meta-analysis. The sample size was 80. After the heterogeneity test (*τ^2 ^= *0.6718; *I^2 ^= *95% > 50%; *P* < 0.05), a random-effect model was used. Meta-analysis results showed that there was a risk (18%) of AE in the combination of IP and SOC (proportion = 0.18; 95% CI, 0.13−0.25). Compared with the incidence of AE in the combination of immunotherapy and SOC (proportion = 0.0773; 95% CI, 0.0589−0.1014), the incidence of AE in the combination of IP and SOC was higher (**Supplementary Figure 37**). A total of two studies reported the occurrence of AE in double arms. The RR value was used to evaluate the side effects of immunotherapy. A sample size was 167. Using random-effect model (*τ^2 ^= *1.0750; *I^2 ^= *91.8% > 50%; *P* < 0.05), meta-analysis results showed that compared with SOC, the incidence of AE of IP combined with SOC increased by 102.91% (RR = 2.0291; 95% CI, 1.2700−3.2418; *Z* = 2.96; *P* = 0.0031 < 0.05) (**Supplementary Figure 38**).

Based on the above results, we summarized the specific AE of immunotherapy (**Table 5**). It was noted that HSV-TK viral therapy resulted in cognitive disorder (RR = 5.08; 95% CI, 0.25−104.76), high intracranial pressure (RR = 5.08; 95% CI, 0.25−104.76), extradural hematomas (RR = 7; 95% CI, 0.37−134.11) and catarrhal symptoms (RR = 11; 95% CI, 0.65−187.42). TGF-2 oligonucleotides suffered from infections (RR = 7.67; 95% CI, 0.41−144.19), brain edema (RR = 6.04; 95% CI, 1.42−25.63), depressed level of consciousness (RR = 12.06; 95% CI, 0.69−211.5), hemiparesis (RR = 12.07; 95% CI, 1.63−89.47), and psychiatric disorder (RR = 5.49; 95% CI, 1.63−89.47). Whereas CpG-ODN led to anemia (RR = 5.38; 95% CI, 0.66−44.07) and sepsis (RR = 9.68; 95% CI, 0.54−174.14).

**Table 5:** Specific Adverse Events of Immunotherapy.

Type of immunotherapy	Symptoms and physical signs.	RR
TGF-β2 oligonucleotides	Infections	7.67[0.41, 144.19]
	Brain edema	6.04[1.42, 25.63]
	Depressed level of consciousness	12.06[0.69, 211.5]
	Hemiparesis	12.07[1.63, 89.47]
	Psychiatric disorder	5.49[0.67, 45.04]
Cpg oligonucleotides	Anaemia	5.38[0.66, 44.07]
	Sepsis	9.68[0.54, 174.14]
HSV-TK viral therapy	Cognitive disorder	5.08[0.25, 104.76]
	Intracranial pressure	5.08[0.25, 104.76]
	Extradural hematoms	7[0.37, 134.11]
	Catarral symptoms	11[0.65, 187.42]

## 4 Discussion

Immunotherapy is playing an increasingly important role in the treatment of tumors. Various types of immunotherapy are available to patients. Thus, clinicians and researchers alike need to understand which types of immunotherapy will be most effective in a given disease setting. To this end, we performed a systematic review of the efficacy and safety of immunotherapy and SOC in adult HGG patients. Our results showed that immunotherapy combined with TMZ yielded better results than SOC alone.

We found that for HGG, the order of efficacy for the immunotherapies evaluated was as follows: DC therapy > VT > IP. DC vaccination triggers *de novo* immune responses against foreign antigens by activating T cells and B cells, which provides a theoretical basis for the development of vaccines against tumor cells. Consistent with our findings, recent results from a large phase III clinical trial (NCT00045968) of an autologous tumor lysate-loaded DC vaccine (DCVax-L) combined with TMZ showed that the median (m)OS was significantly extended in both newly diagnosed patients and those with recurrent GBMs; the therapy also had a good safety profile (25, 26). Notably, because of pseudoprogression and the fact that placebo patients received DCVax-L following crossover, OS, rather than PFS, was considered a feasible endpoint, by comparison to external control populations. The mOS of newly diagnosed GBM patients in the DCVax-L group (N = 232) was 19.3 months (95% CI, 17.5–21.3) from randomization vs. 16.5 months (95% CI, 16.0–17.5) in the control cohort (N = 1,366). For patients with recurrent GBM, mOS was 13.2 months (95% CI, 9.7–16.8) from relapse for patients receiving DCVax-L (N = 64) and 7.8 months (95% CI, 7.2–8.2) for the control group (N = 640). Besides, a meta-analysis also assessed the clinical impact of DC vaccination and VT in comparison to SOC for patients with HGG (27). Eight phase I/II clinical trials of DC vaccines were analyzed and the results showed that OS was markedly improved in both patients with newly diagnosed (HR = 0.65) and recurrent (HR = 0.63) HGG when treated a DC vaccine vs. SOC; however, improvement in PFS was not statistically significant (*P* = 0.1), which is consistent with our findings. Another meta-analysis performed by Lv et al. included data from six phase II RCTs of DC vaccines in patients with GBM and reported that OS was significantly improved following treatment with a DC vaccine vs. placebo or blank treatment (HR = 0.69; 95% CI, 0.49–0.97; *P* = 0.03 < 0.05) (28). In this case, however, PFS in GBM patients was somewhat improved as a result of DC vaccination (HR = 0.76; 95% CI, 0.56–1.02; *P* = 0.07), without significant heterogeneity (*I²* = 0). However, the preparation methods and activation strategy of the DC vaccines differed between studies. Future research needs to determine whether the preparation and activation mode of the DC vaccine affects the efficacy of this immunotherapy.

Our analysis of oncolytic VT trial data showed that, compared with SOC, the combination of VT and SOC did not provide any statistically significant improvement in OS or PFS for patients with HGG. The VT injection specific characteristics were shown here (**Table 6**). Only the results of a subgroup analysis indicated that VT prolonged OS with optimized injection methods. In accordance, a meta-analysis of four phase I/II/III clinical trials reported that VT did not significantly improve the OS or PFS of patients with newly diagnosed HGGs (27). Currently, there are more than 20 trials registered at *ClinicalTrials.gov* evaluating the efficacy and safety of oncolytic VT in patients with glioma. The modified viral species associated with encouraging results in phase I/II clinical trials include herpes simplex virus, reovirus, vaccinia virus, adenovirus and parvovirus. Although it did not meet the inclusion criteria of the present study, a recent phase II clinical trial evaluated the survival benefits and safety profile of immunotherapy with an intratumoral, oncolytic herpes virus, G47Δ, in residual or recurrent GBM (29). The 1-year survival rate of G47Δ-treated patients was 84.2% (95% CI, 60.4–96.6; 16 of 19) and the mOS was 20.2 months (16.8–23.6 months) vs. 28.8 months (20.1–37.5 months) for patients treated with initial surgery alone. Moreover, patients treated with G47Δ had higher a number of tumor-infiltrating CD4^+^ and CD8^+^ T lymphocytes and persistently lower Foxp3 levels in the tumor tissues, than controls. These promising results led to the approval of G47Δ in Japan as the first oncolytic VT product to be used for the treatment of patients with malignant glioma.

**Table 6:** Specific characteristics of viral therapy injections.

Name	Volume	Dose	Cycle	Course	Injection
Desjardins 2018	3.5ml	10^8^~10^10^	1	6.5 hours	/
Immomen 2004	10ml	3x10^10^	1	/	30-70 injections
Ji 2016	/	1x10^12^	≥2	21 Days	/
Rainov 2000	9-10ml	1x10^8^	1	/	/
Westphal 2013	10ml	1x10^12^	1	/	/
Wheeler 2016	1ml	3x10^11^	1	/	10 injections

/, Not available.

For the IP, two trials were analyzed in our study (20, 21). CpG-ODN is a new type of immunostimulating agent, which comprises an immunomodulatory synthetic ODN specifically designed to stimulate Toll-like receptor 9 (30). Currently, there are four phase I/II clinical trials of CpG-ODN involving glioma patients, one of which met our study criteria (21). The study found that IP exhibited poor efficacy, which the authors claimed was unexpected and may have been related to the selection bias of the enrolled patients with recurrent GBM or a difference in the mode of CpG-ODN administration. In accordance, a phase II clinical trial of CpG-ODN, administered intracerebrally to patients with recurrent GBM, did not meet the targeted PFS; however, encouragingly, a few long-term survivors were observed (31). Another IP currently in use is trabedersen (also known as OT-101). It is a synthetic antisense ODN designed to block the production of human TGF-β2. TGF-β2 is reported to exert protumor effects in the tumor microenvironment (TME) *via* different mechanisms, such as by stimulating angiogenesis, promoting T cell exclusion, and preventing helper T (Th)1 effector phenotype differentiation, which collectively contribute to tumor immune evasion (32). A recent *post-hoc* analysis of a phase II clinical trial (NCT00431561) data was performed to evaluate the efficacy of trabedersen treatment for recurrent and/or refractory HGG with a poor prognosis (33). The results showed that the intratumorally delivery of trabedersen, *via* a convection-enhanced delivery system, exhibited promising single-agent antitumor activity, resulting in a PFS of > 3 years and an OS of > 3.5 years in over a third of HGG patients. Notably, pseudoprogression was observed in ~10% of patients receiving trabedersen, which was associated with improved survival. To date, only one phase III clinical trial (NCT00761280, initiated in 2008) of trabedersen has involved glioma patients; unfortunately, it was terminated early in 2012 because it did not fulfil its projected patient recruitment figures. Future clinical trials are therefore warranted to further evaluate the efficacy and safety profiles of IP in large-scale patient cohorts.

Due to our stringent criteria, only DC therapy, VT, and IP met the requirements and were included in the study. Notwithstanding, other types of immunotherapy, such as ICIs and CAR (either T or natural killer [NK] type), have shown promise in the treatment of glioma. ICI therapy is one of the earliest forms of cancer immunotherapy. It functions by restoring cytotoxic T cell activity and enhancing antitumoral adaptive immunity (34). Glioma cells express high levels of immunosuppressive factors, such as programmed cell death receptor 1 (PD-1) and cytotoxic T lymphocyte-associated antigen-4 (CTLA-4), which reduce the proliferation and activation ability of T cells and weaken the antitumor immune response. Antibodies against CTLA-4, PD-1, or its ligand, PD-L1, are the most widely studied ICIs in the clinic (35). The success of ICIs in treating various solid tumors has aroused great interest in relation to their application to treat brain tumors (36, 37). Although no ICI trials met our study inclusion criteria, a study that retrospectively analyzed 66 GBM patients who were treated with SOC and the PD-1 inhibitors, pembrolizumab or nivolumab, confirmed that the OS of patients who were responsive to immunotherapy was significantly longer than that of the non-responders (14.3 vs. 10.1 months) (38). Further genomic and transcriptome profiling also revealed multiple genomic alterations and evolutionary patterns in GBM patients undergoing anti-PD-1 immunotherapy. These included enrichment in MARK pathway changes in responders and the increased *PTEN* mutations, which correlated with immunosuppressive expression characteristics, in non-responders. Of note, the CheckMate 143 trial, which was the first randomized phase III study of an ICI in patients with primary brain tumors (NCT02017717) (39), evaluated the efficacy and safety of nivolumab (a PD-1 inhibitor) alone or in combination with bevacizumab (a vascular endothelial growth factor [VEGF] inhibitor) in patients with first relapsed GBM following standard radiotherapy and TMZ treatment. A total of 369 patients were randomized to nivolumab (n = 184) or bevacizumab (n = 185) and no statistically significant difference was found in the risk of death between the groups after treatment (HR = 1.04; 95% CI, 0.83–1.30; *P* = 0.76). However, a subgroup analysis showed that patients with a methylated *MGMT* promoter and no baseline corticosteroid use may potentially benefit from ICI treatment. Besides, clinical trials are currently underway to evaluate the use of local radiotherapy in combination with anti-PD-1 antibodies in patients with newly diagnosed or recurrent GBM (NCT02648633 and NCT02866747). Currently, the clinical benefits of new immune checkpoint molecules, such as inhibitors of the V-domain immunoglobulin suppressor of T cell activation (VISTA), CD73, and CD38, are being studied. Despite the fact that ICI therapies have improved patient outcomes across numerous cancer types, only a minority (< 10%) of GBM patients achieve a durable response (38, 40, 41). Importantly, current ICI treatment regimens are usually maintained for ~2 years; whether longer-term ICI treatment can improve the curative effect is still under study. Thus, more preclinical and clinical research is needed to further verify the efficacy of ICIs and explore the mechanism underlying their failure in the treatment of HGGs, including GBM.

CAR T cell therapy has shown promising therapeutic effects in patients with hematological malignancies; however, its application in the treatment of GBM is still in the early stages of development. A preliminary study by Brown et al. evaluated the effect of repeated intracranial injection of CD8^+^ CAR T cells targeting the interleukin (IL)-13 receptor subunit alpha 2 (IL-13Rα2), which is overexpressed in > 50% of GBMs, in three patients with relapsed GBM (42). The treatment was well tolerated and transient antitumor activity was seen in two-thirds of the patients. However, the expression of IL-13Rα2 in residual tumor tissue adjacent to the injection site was significantly reduced, implying that antigen loss occurred as a result of treatment. To address this issue, new CAR T strategies targeting IL-13Rα2 were developed and evaluated in several preclinical and phase I clinical studies (43–45). Notably, the regression of intracranial tumor and spinal metastasis were observed in a patient with recurrent GBM after treatment with IL13Rα2-targeting CAR T cells; moreover, the patient’s clinical response lasted for 7.5 months (43). The selection of specific T cell subsets is one of the approaches being used to improve CAR T cell therapy and optimize antitumor efficacy. CD8^+^ T cells have long been the primary cell population used to develop CAR T cell therapies to treat brain tumors. A recent study compared the antitumor effect of CD8^+^ and CD4^+^ CAR T cells targeting IL-13Rα2 in GBM. The authors found that although CD8^+^ CAR T cells exhibited a potent short-term effect, they were prone to rapid exhaustion, while the CD4^+^CAR T cell-mediated long-term antitumor response outperformed that of the CD8^+^ CAR T cells (46). This result demonstrates that CD4^+^ T cells are an important alternative T cell subset for effective CAR therapy.

In preclinical studies, CAR T cells targeting the epidermal growth factor receptor (EGFR)vIII were efficiently delivered to tumor sites and inhibited the growth of glioma xenografts in murine models (47). EGFRv III is a variant of the EGFR, which is expressed in ~30% of GBM patients and is associated with a poor prognosis. Ten patients with recurrent GBM were adoptively transferred CAR T cells, which were transported in peripheral blood to the intracranial tumor sites, where they exerted an antitumor effect. Interestingly, analysis of pre- and post-treatment tumor samples revealed a decrease in tumor antigen expression and an increase in the presence of inhibitory immune checkpoint molecules and regulatory T cells at the tumor site after treatment, indicating increased tumor resistance (48); other clinical trials are currently underway to further evaluate the efficacy of these CAR T cells. Human epidermal growth factor receptor 2 (HER2), a receptor tyrosine kinase overexpressed in up to 80% of GBMs, has also recently been recognized as an ideal tumor-associated antigen for CAR targeting in GBM (49, 50). Seventeen patients underwent a phase I trial with peripheral blood infusions of virus-specific T cells modified with a HER2-specific CAR (51). The HER2-CAR T cells did not proliferate but persisted at a low frequency for up to 1 year. Of the 16 evaluable patients, one patient had a partial response lasting more than 9 months, while seven patients had stable disease lasting between 8 weeks and 29 months (three of whom had no progression between the 24- and 29-month follow-up timepoints).

Despite the promising efficacy, the manufacturing time, cost, and in particular, the severe toxicities (e.g., neurotoxicity, immune-effector-cell-associated neurological syndrome, and cytokine release syndrome) associated with CAR T cell therapy highly limit its application. NK cells are a group of unique antitumor effector cells, which have the functions of cytotoxicity, cytokine production, and immune memory, but unlike T cells, are not limited by major histocompatibility complex (MHC)-mediated antigen recognition (52). The favorable safety profile and antitumor potential of NK cells make them promising cells for the implementation of CAR technology. In addition, because NK cells do not require MHC restriction, CAR NK cells can be generated a lot more rapidly than CAR T cells. However, the efficacy of CAR NK therapy needs to be clinically tested and challenges such as the low persistence of NK cells *in vivo* and their limited proliferative potential have to be overcome (53).

Nonetheless, given existing challenges in HGG, such as the high tumor heterogeneity, protumor and anti-inflammatory TME, and blood-brain barrier, patients with HGG are unlikely to benefit from mono-immunotherapy. Recent studies suggest that immunotherapy is an exciting candidate for combination therapy in HGG and many clinical trials are underway to explore suitable combinational strategies. For instance, a single-arm phase II clinical trial (NCT02550249) showed that the use of nivolumab as a neoadjuvant therapy in patients with GBM undergoing surgery resulted in the modulation of the TME (e.g., increased immune cell infiltration and broader T cell receptor [TCR] clonal diversity among the tumor-infiltrating T lymphocytes) (40). Radiotherapy and chemotherapy increase the efficacy of immunotherapy *via* multiple mechanisms, for instance by modulating the TME, increasing the expression and presentation of tumor antigens, and eliminating immunosuppressive cells (54). Results from some preclinical models suggest that chemotherapy can be synergistically used in combination with CpG-ODN to treat tumors, including gliomas. The main reason for this is the abscopal effect caused by radiotherapy and chemotherapy. Radiotherapy and chemotherapy induce the apoptosis of tumor cells. The lysed tumor cells release a large number of immunogenic substances. These substances activate immune cells, thereby triggering a more effective antitumor immune response (55–58). Combining different types of immunotherapy is also considered a promising strategy for HGG treatment (59). For example, ICIs have been proven to improve the antitumor effect of CAR T cell therapy in preclinical studies (60); this combinatory therapy has recently reached the clinical setting for the treatment of GBM (NCT03726515, NCT04003649). The combination of VT and CAR T cell therapy has also shown a synergistic effect by improving survival and tumor regression in a mouse model (61). Besides, the efficacy and safety of DC vaccination combined with ICIs are also being evaluated in phase I/II clinical trials involving GBM patients (NCT03422094, NCT04013672).

Our study has some limitations. First, because the number of incorporated studies was small, our conclusion regarding the efficacy and safety of immunotherapy in the context of glioma should be interpreted with caution. Second, the subgroups based on immunotherapy type were divided into open-label and double-blind groups. However, only one trial was included in the double-blind group. This was mainly due to the insufficient number of studies; we hope that more double-blind trials will be conducted in the future. Third, in the study by Ji et al., the OS of patients in the control arm was only 2.0–3.3 months (17), which was shorter than that reported by all other trials. This short OS may be related to the social attitudes and/or medical conditions in China, whereby a delay to treatment initiation may be caused by the negative connotations associated with seeking medical treatment early and/or barriers to accessing medical treatment. Fourth, we found that glioma patients from China benefited the most from immunotherapy, compared to patients from other countries evaluated. It is possible that racial and/or regional lifestyle differences are also important influencing factors.

In summary, we believe that immunotherapy will become increasingly important in the treatment of patients with glioma, and we hope that it will be considered by clinicians as an adjuvant therapy to chemotherapy and radiotherapy. Besides, our results showed that the efficacy of immunotherapy could be improved by addressing the associated safety concerns. For instance, the IP-mediated stimulation of the innate immune system elicits strong side effects. Thus, when scientists develop new agents for modulating the innate immune system, their safety index should be considered. In addition, we found that DC vaccines were typically injected intradermally into the axilla rather than intracranially, which was a safer method of administration than that used in VT. Thus, our findings indicate that the efficacy and safety of VT may be influenced by injection methods. These methods could not only be optimized by administering multiple courses of treatment, performing multi-point injections, or using small injection volumes, but also by changing to a new genetic vector, such as liposome-, polymer-, or protein-based delivery systems.

## Data availability statement

The original contributions presented in the study are included in the article/**Supplementary Material**. Further inquiries can be directed to the corresponding authors.

## Author contributions

BG, SZ conceptualized formal analysis and screened literature and assessed risk of bias. LX and W-LC took part in revision and writing. JS and PZ was responsible for statistical analysis and screened literature. JZ and LZ provided with instructions on methodology. All authors contributed to the article and approved the submitted version.
