# Supplementary material for: Efficacy and safety of innate and adaptive immunotherapy combined with standard of care in high-grade gliomas: a systematic review and meta-analysis
Source: Front Immunol. 2023 Jul 6;14:966696. doi: 10.3389/fimmu.2023.966696 (PMC10357294; doi:10.3389/fimmu.2023.966696)
Supplement: Supplementary file 1 [file DataSheet_1.docx]

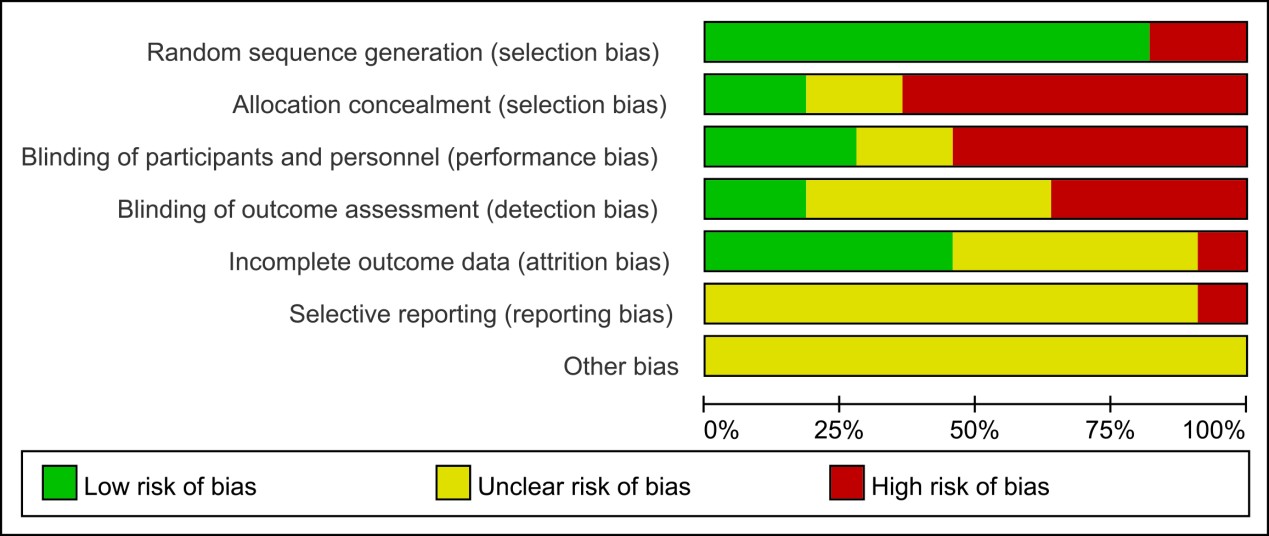


Supplementary Figure 1. Risk of bias summary in the included study


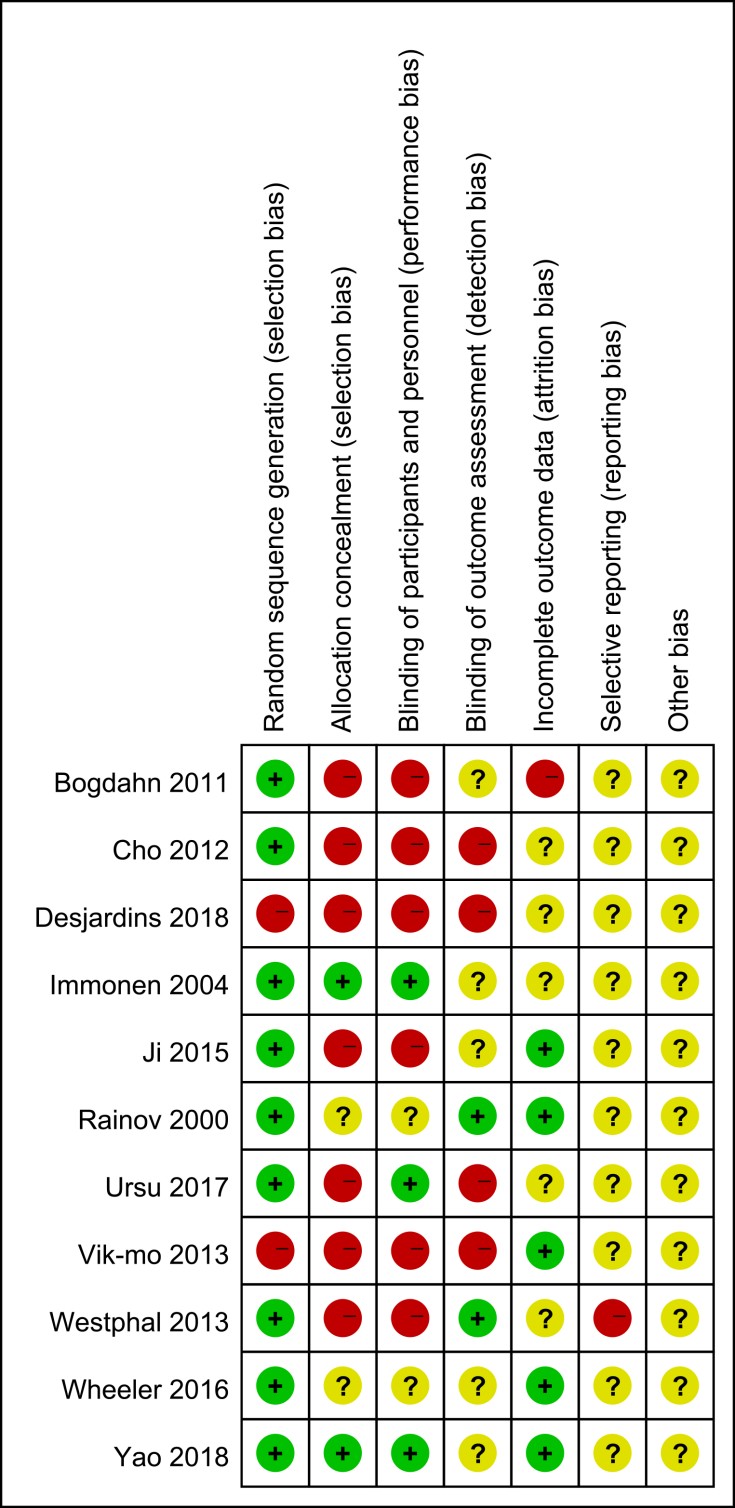


Supplementary Figure 2. Risk of bias graph in the included study


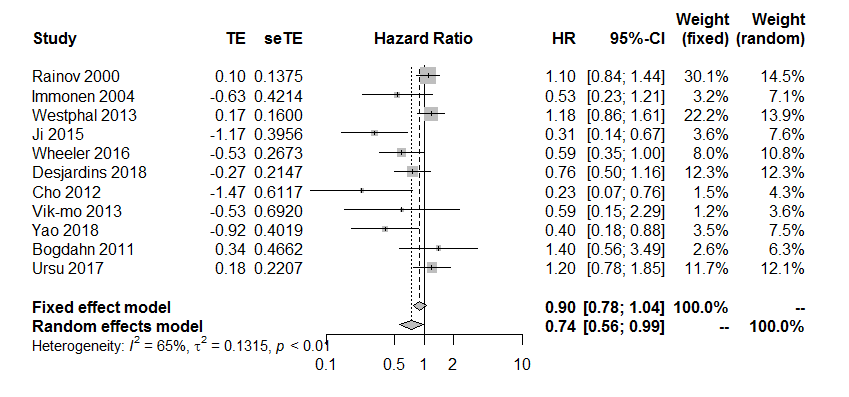


Supplementary Figure 3. Analysis of the OS of the combination of immunotherapy and standard of care compared with standard of care


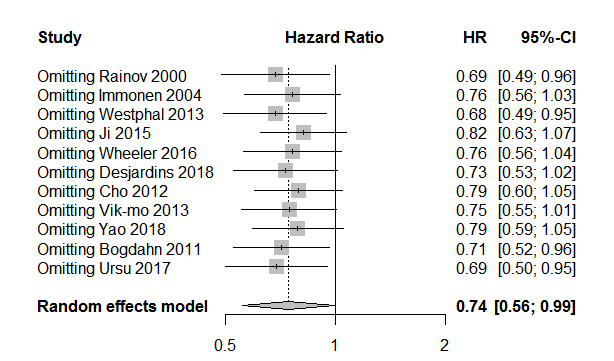


Supplementary Figure 4. Sensitivity analysis of the combination of immunotherapy and standard of care compared with standard of care according to OS


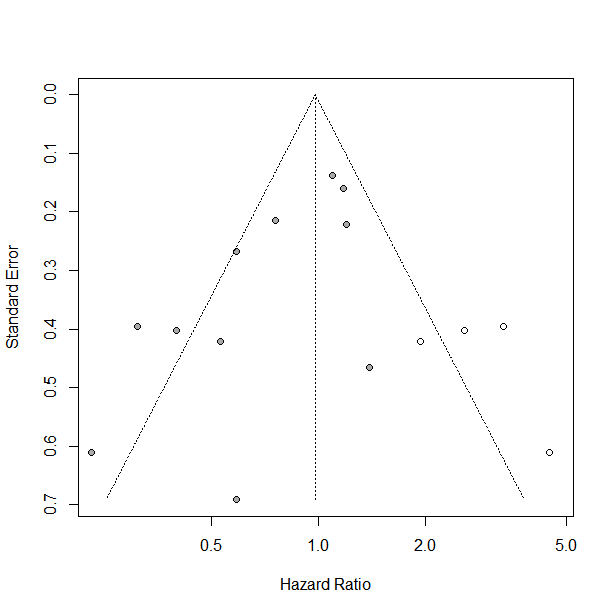


Supplementary Figure 5. Publication bias of the combination of immunotherapy and standard of care compared with standard of care according to OS





Supplementary Figure 6. Subgroup analysis of the combination of immunotherapy and standard of care compared with standard of care according to clinical tumor type


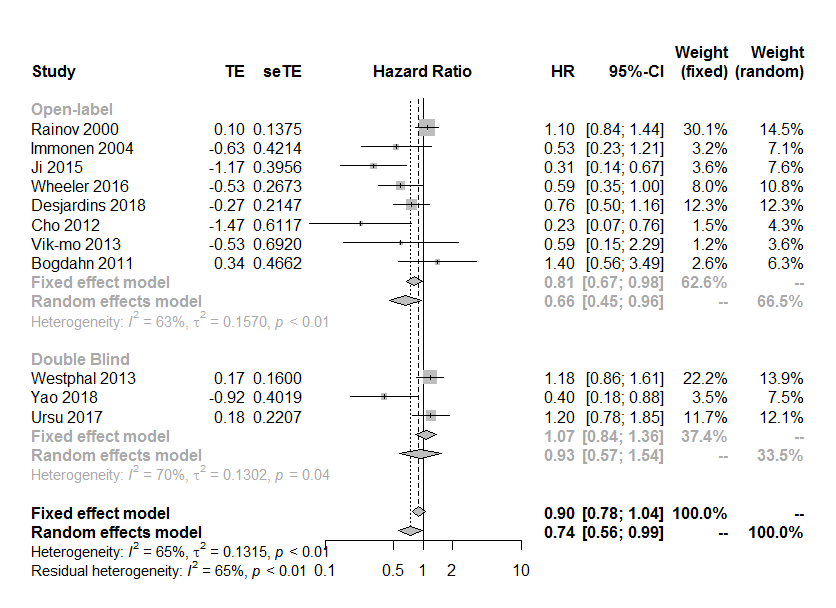


Supplementary Figure 7. Subgroup analysis of the combination of immunotherapy and standard of care compared with standard of care according to blind method
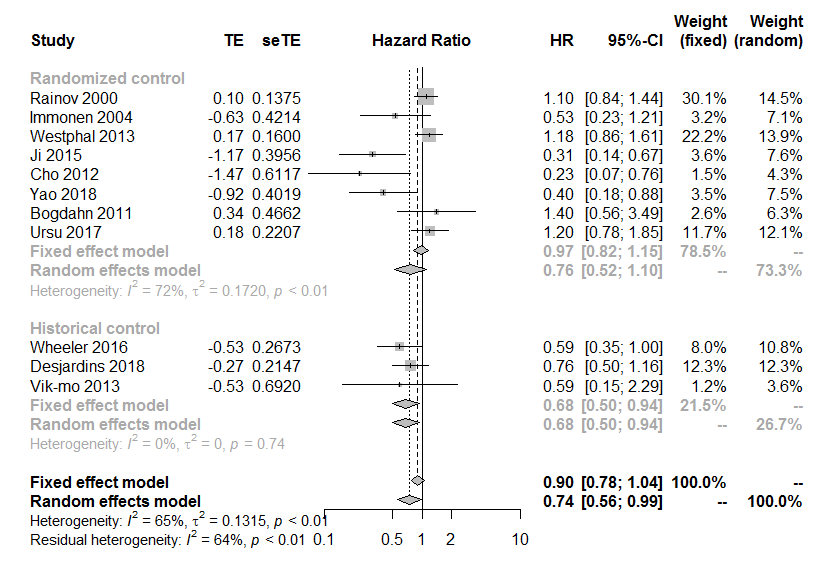


Supplementary Figure 8. Subgroup analysis of the combination of immunotherapy and standard of care compared with standard of care according to allocation method


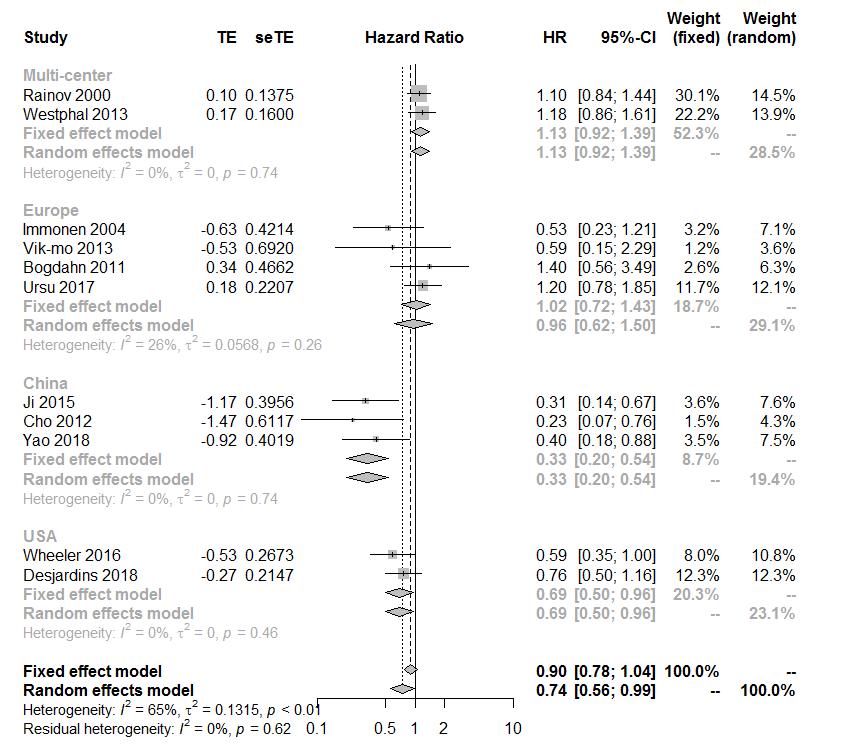


Supplementary Figure 9. Subgroup analysis of the combination of immunotherapy and standard of care compared with standard of care according to recruiting area


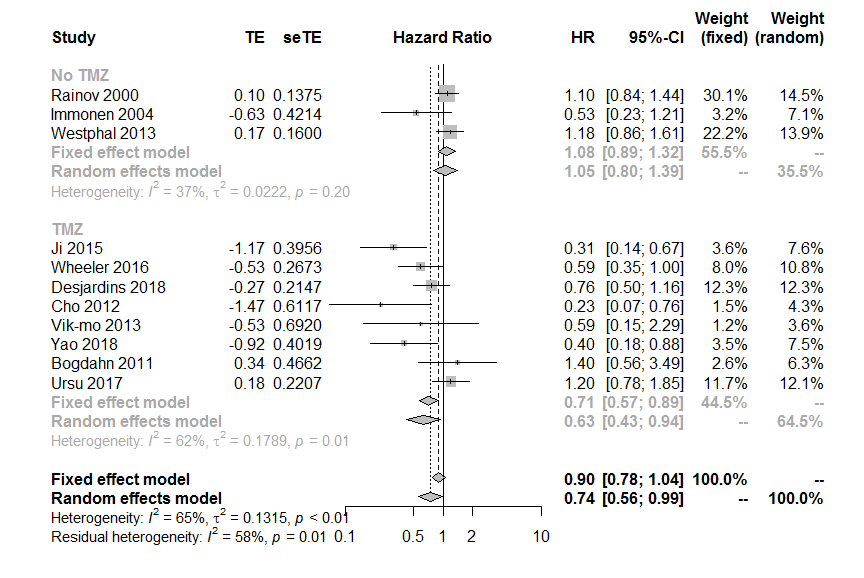


Supplementary Figure 10. Subgroup analysis of the combination of immunotherapy and standard of care compared with standard of care according to the usage of TMZ


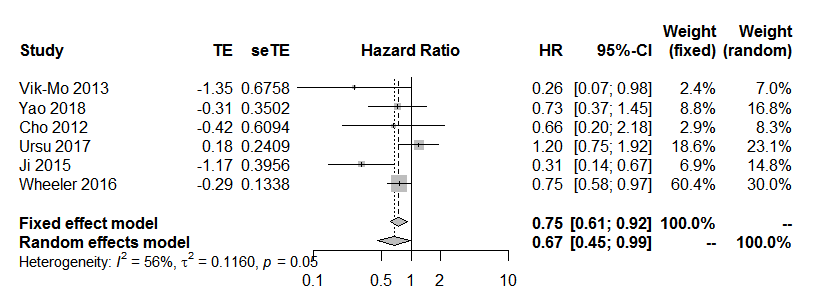


Supplementary Figure 11. Analysis of the PFS of the combination of immunotherapy and standard of care compared with standard of care


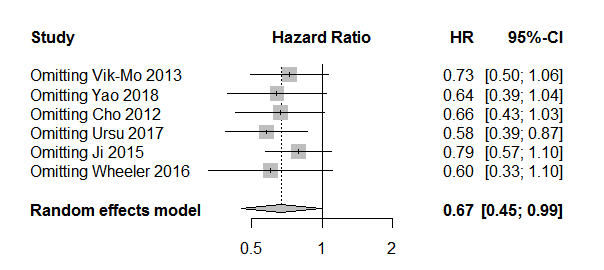


Supplementary Figure 12. Sensitivity analysis of the combination of immunotherapy and standard of care compared with standard of care according to PFS


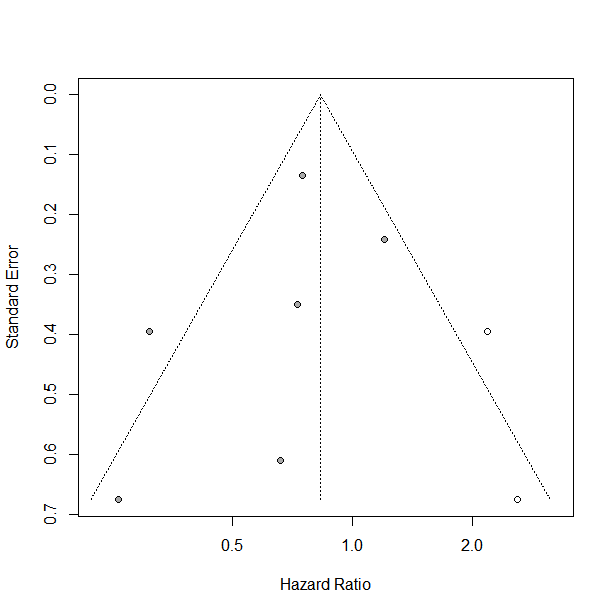


Supplementary Figure 13. Publication bias of the combination of immunotherapy and standard of care compared with standard of care according to PFS


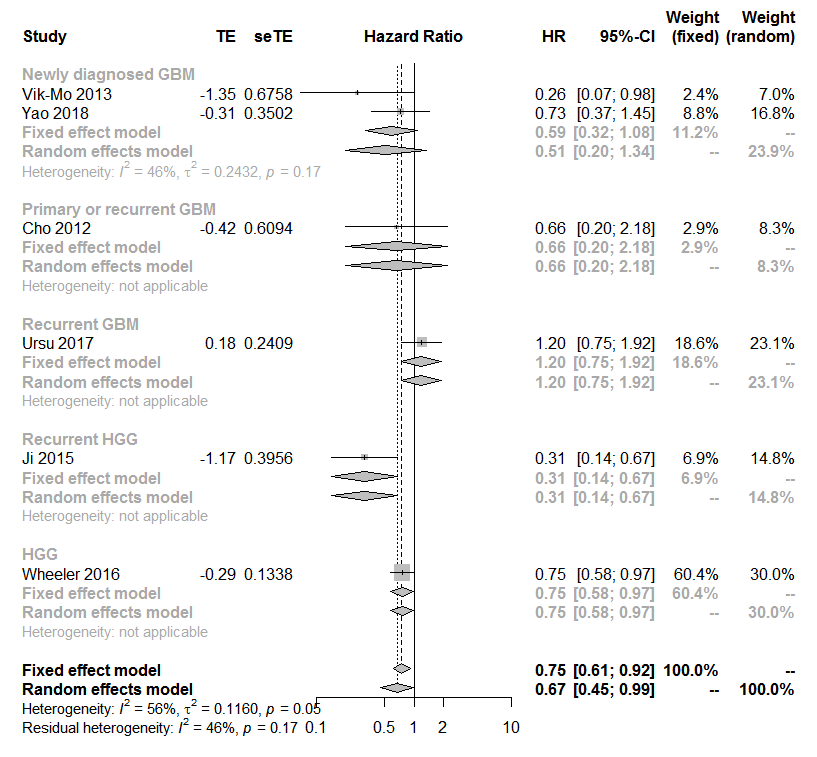


Supplementary Figure 14. Subgroup analysis of the combination of immunotherapy and standard of care compared with standard of care according to clinical lesion type


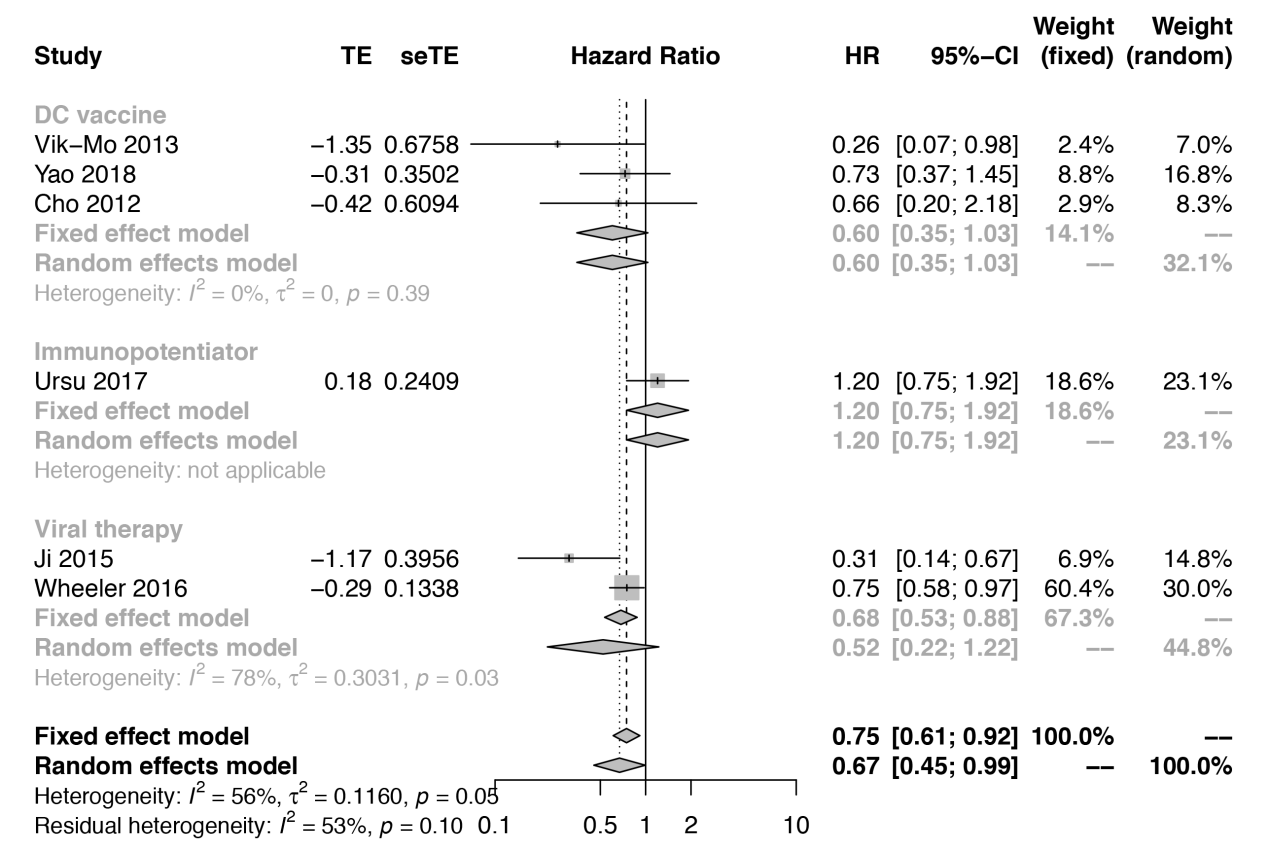


Supplementary Figure 15. Subgroup analysis of the combination of immunotherapy and standard of care compared with standard of care according to therapeutic scheme


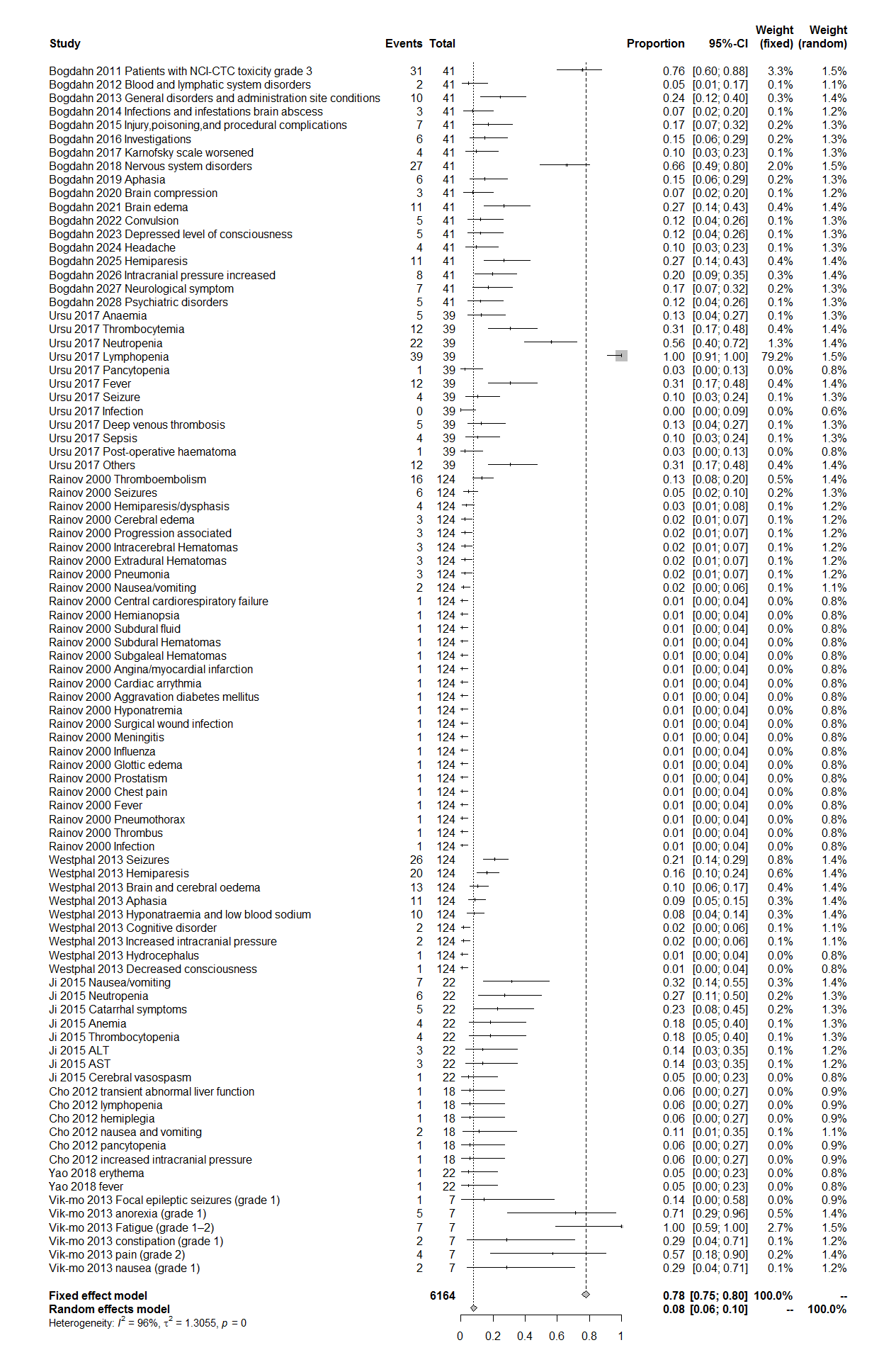


Supplementary Figure 16. Analysis of the incidence of adverse events of the combination of immunotherapy and standard of care


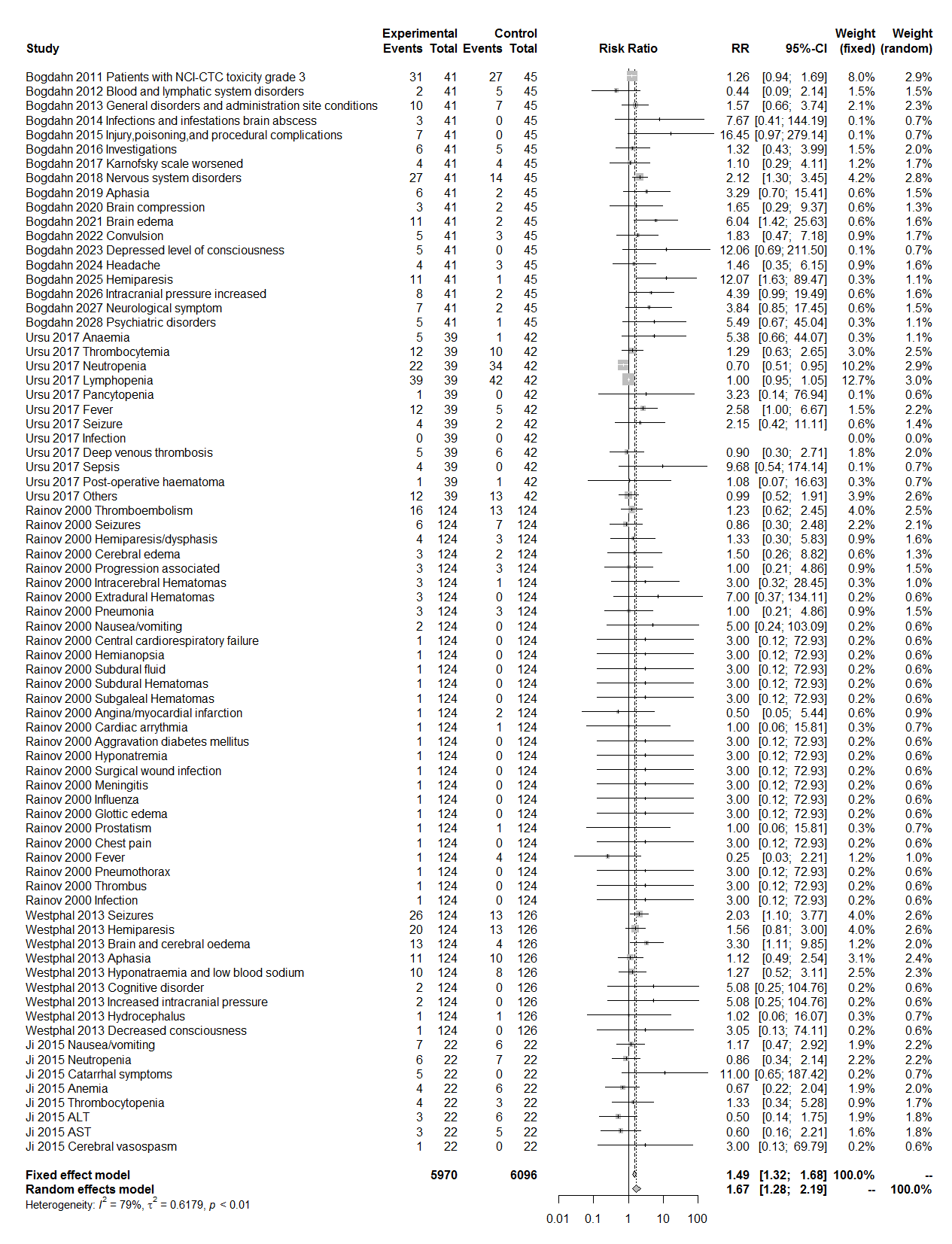


Supplementary Figure 17. Analysis of the risk ratio of adverse events of the combination of immunotherapy and standard of care compared with standard of care


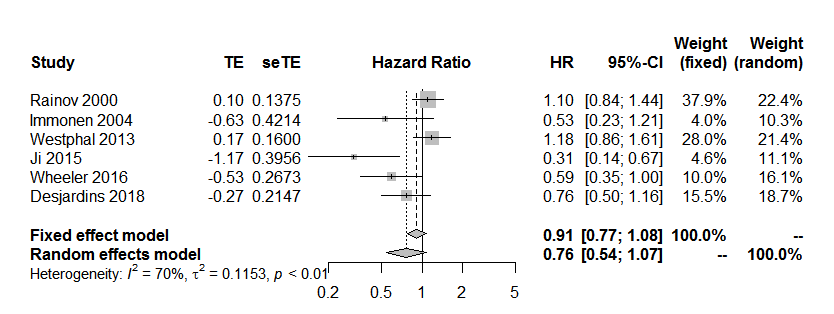


Supplementary Figure 18. Analysis of the OS of the combination of viral therapy and standard of care compared with standard of care


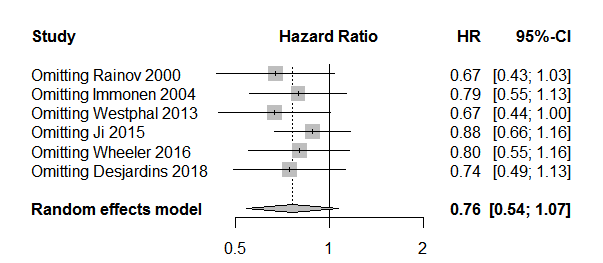


Supplementary Figure 19. Sensitivity analysis of the combination of viral therapy and standard of care compared with standard of care according to OS


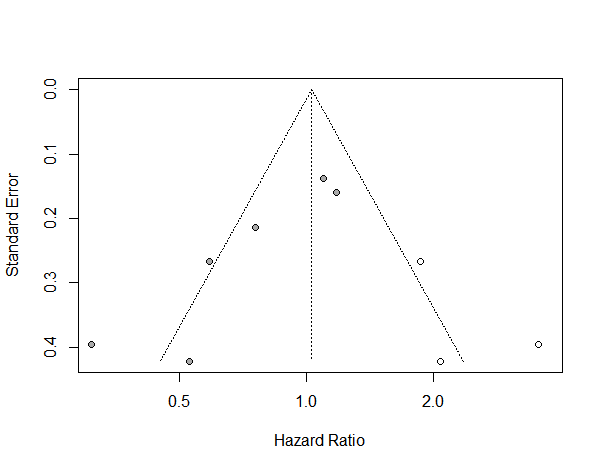


Supplementary Figure 20. Publication bias of the combination of viral therapy and standard of care compared with standard of care according to OS


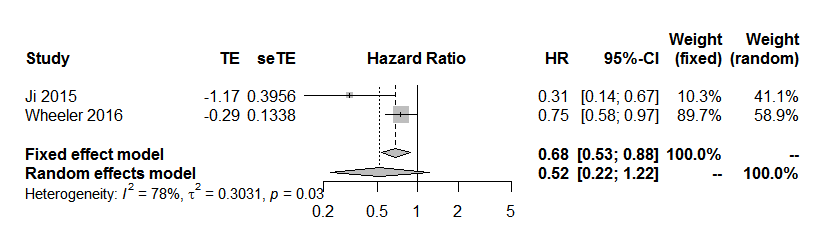


Supplementary Figure 21. Analysis of the PFS of the combination of viral therapy and standard of care compared with standard of care


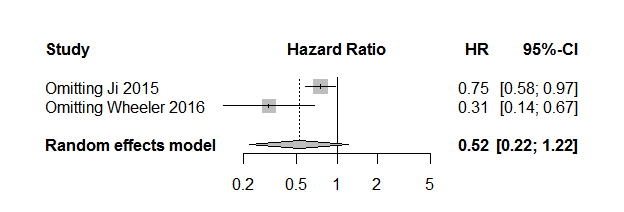


Supplementary Figure 22. Sensitivity analysis of the combination of viral therapy and standard of care compared with standard of care according to PFS


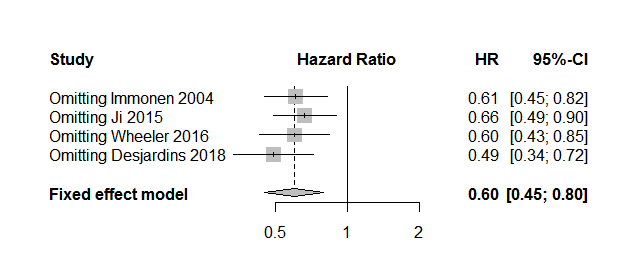


Supplementary Figure 23. Sensitivity analysis of the combination of multiple courses of treatment/multi-point injection/ small injection volume viral therapy and standard of care compared with standard of care according to OS


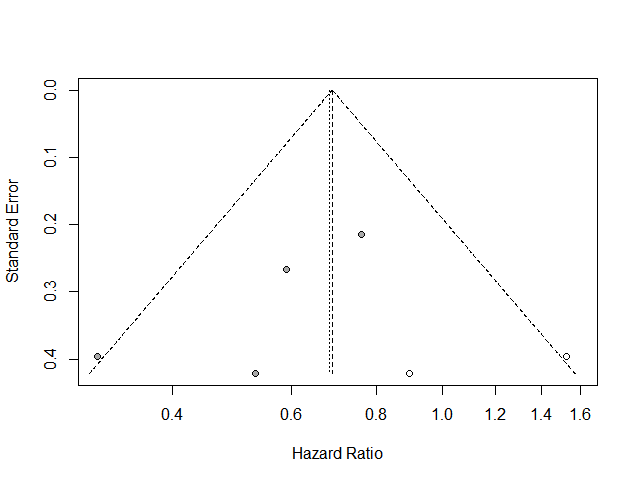


Supplementary Figure 24. Publication bias of the combination of multiple courses of treatment/multi-point injection/ small injection volume viral therapy and standard of care compared with standard of care according to OS


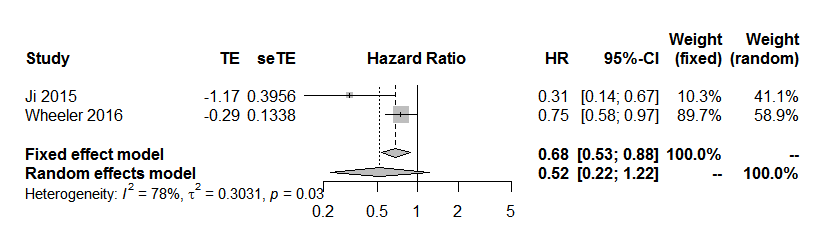


Supplementary Figure 25. Analysis of the PFS of the combination of multiple courses of treatment/multi-point injection/ small injection volume viral therapy and standard of care compared with standard of care


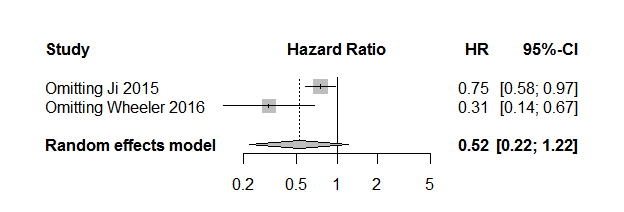


Supplementary Figure 26. Sensitivity analysis of the combination of multiple courses of treatment/multi-point injection/ small injection volume viral therapy and standard of care compared with standard of care according to PFS


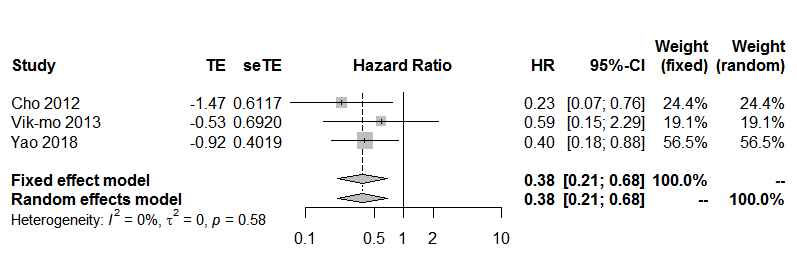


Supplementary Figure 27. Analysis of the OS of the combination of DC therapy and standard of care compared with standard of care


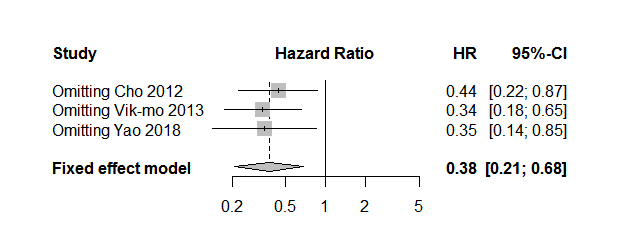


Supplementary Figure 28. Sensitivity analysis of the combination of dendritic cell vaccine and standard of care compared with standard of care according to OS


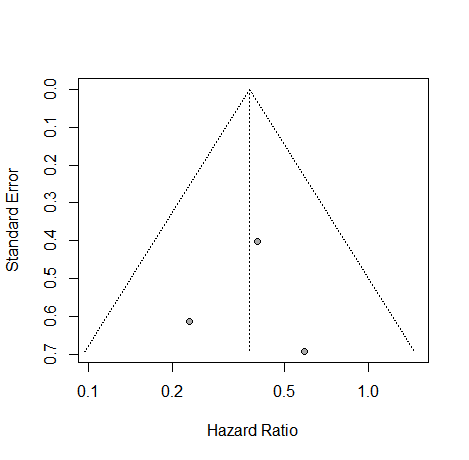


Supplementary Figure 29. Publication bias of the combination of multiple courses of DC therapy and standard of care compared with standard of care according to OS


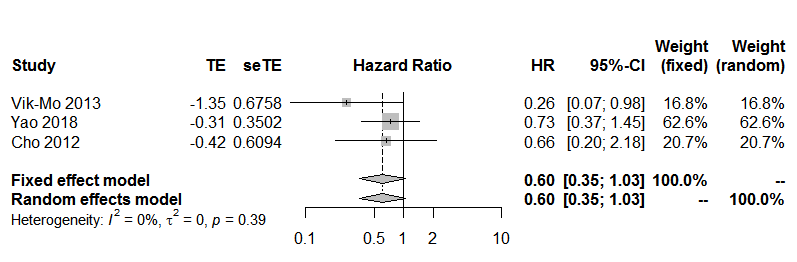


Supplementary Figure 30. Analysis of the PFS of the combination of DC therapy and standard of care compared with standard of care


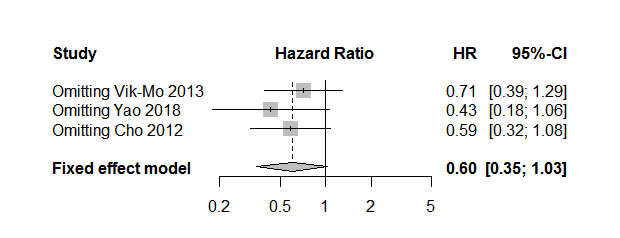


Supplementary Figure 31. Sensitivity analysis of the combination of DC therapy and standard of care compared with standard of care according to PFS


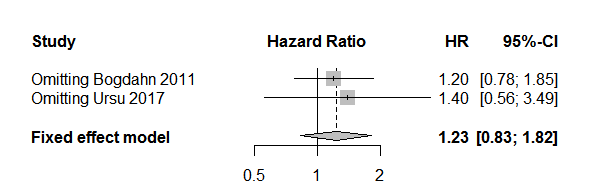


Supplementary Figure 32. Sensitivity analysis of the combination of immunopotentiators and standard of care compared with standard of care according to OS


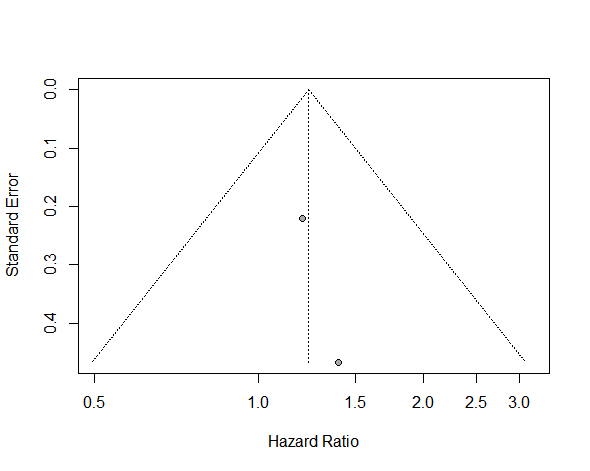


Supplementary Figure 33. Publication bias of the combination of immunopotentiators and standard of care compared with standard of care according to OS


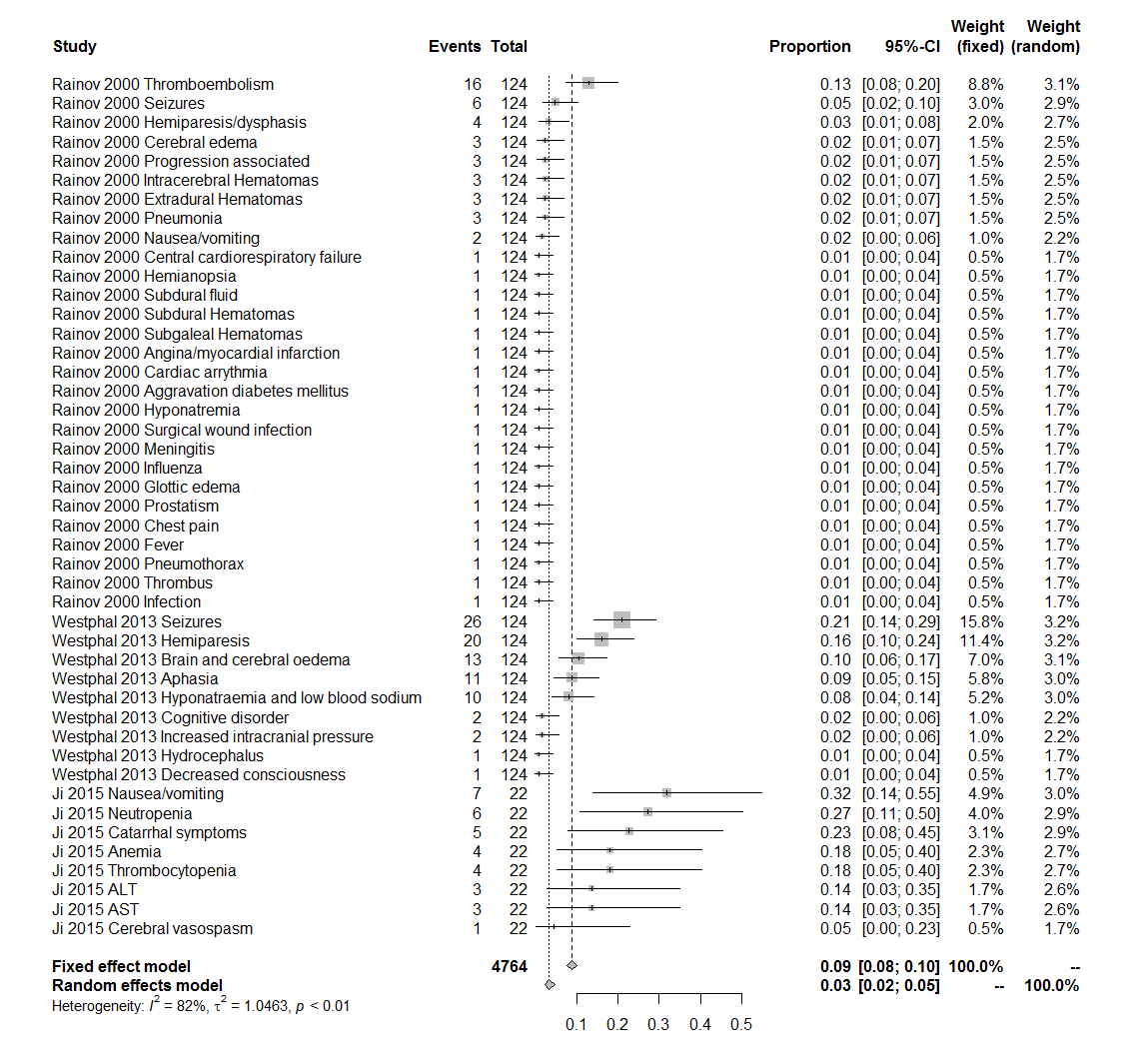


Supplementary Figure 34. Analysis of the incidence of adverse events of the combination of viral therapy and standard of care compared with standard of care


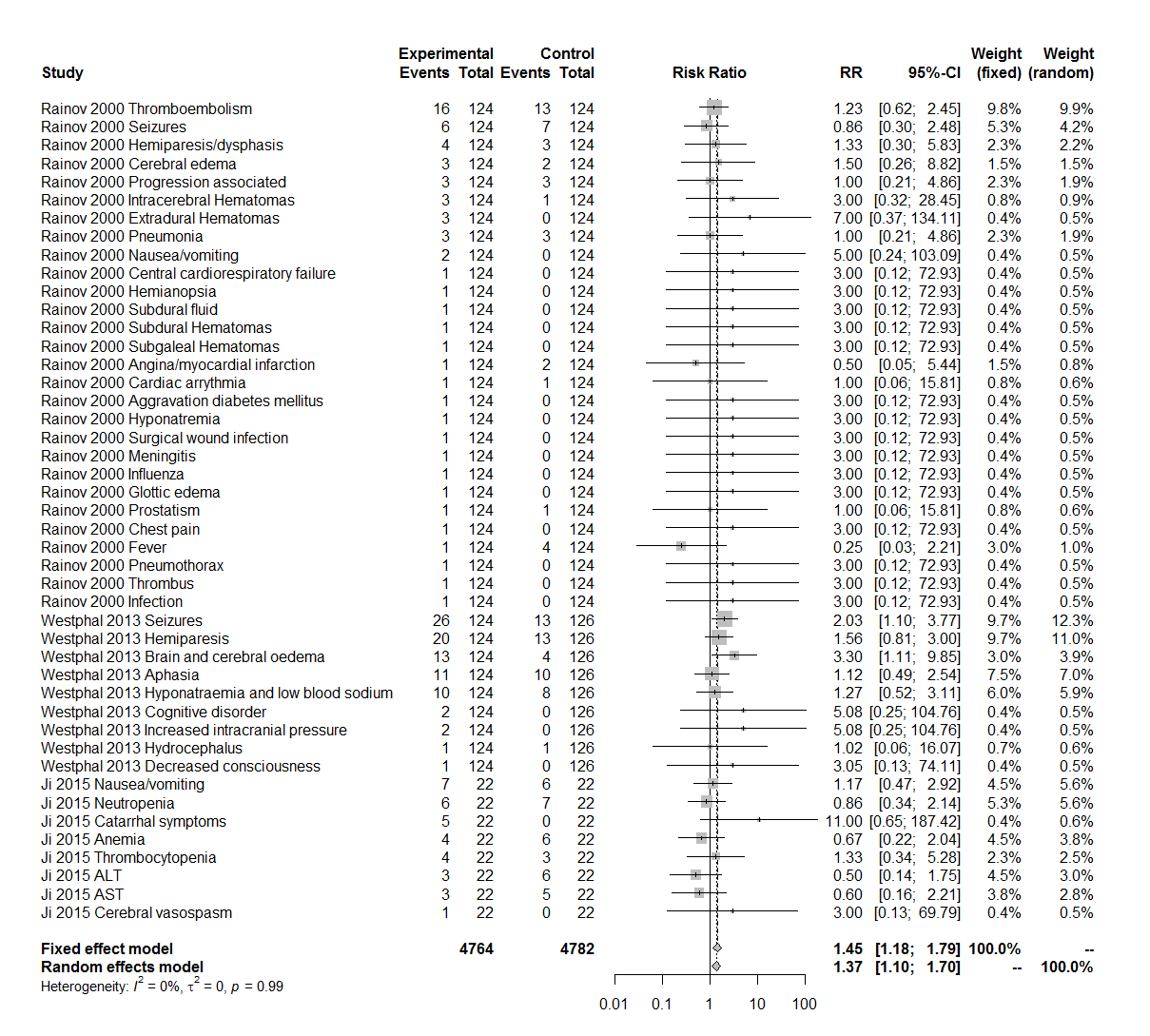


Supplementary Figure 35. Analysis of the risk ratio of adverse events of the combination of viral therapy and standard of care compared with standard of care


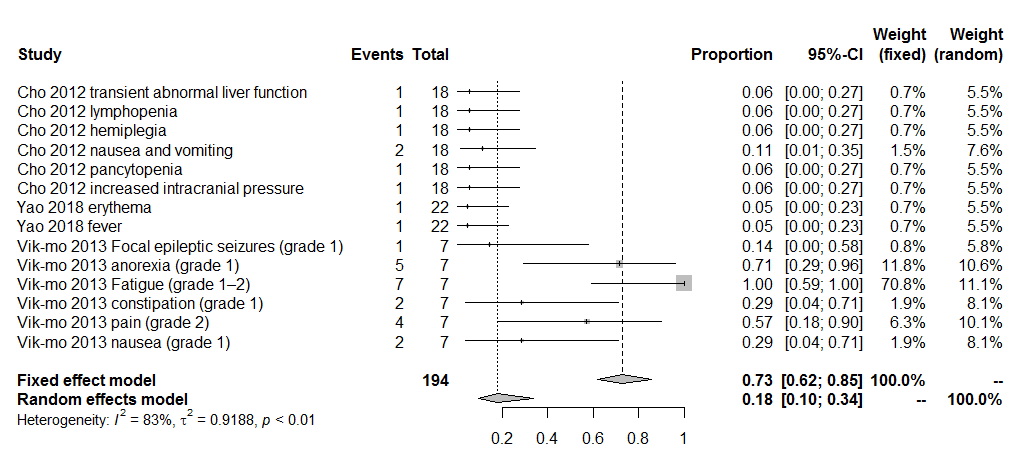


Supplementary Figure 36. Analysis of the incidence of adverse events of the combination of DC therapy and standard of care compared with standard of care


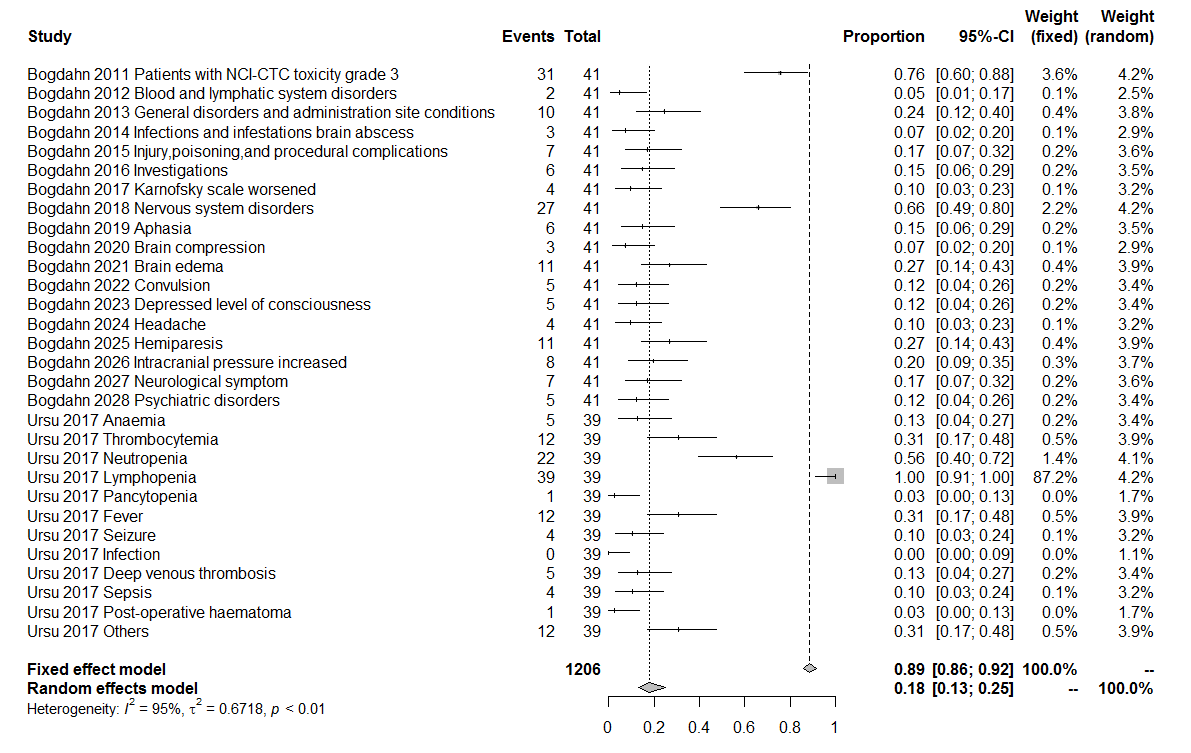


Supplementary Figure 37. Analysis of the incidence of adverse events of the combination of immunopotentiators and standard of care compared with standard of care


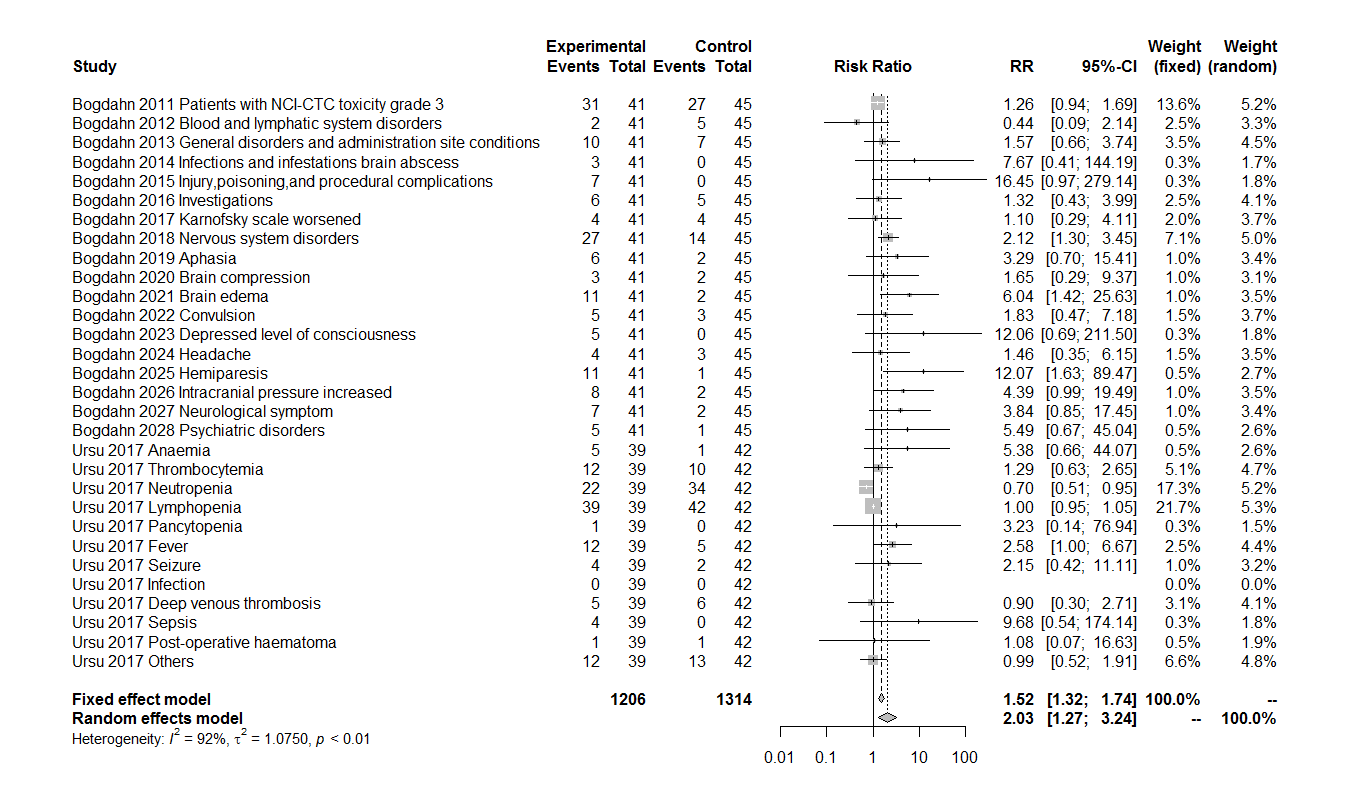


Supplementary Figure 38. Analysis of the risk ratio of adverse events of the combination of immunopotentiators and standard of care compared with standard of care

| Name | Object | Subject | Outcomes | Conclusion |
| --- | --- | --- | --- | --- |
| Hanaei 2018(Hanaei et al., 2018) | Immunotherapy | glioma | Mean OS；1-，2-，3-year OS HS；1-year PFS HR；CR | Immunotherapy could prolong OS but not PFS |
| Artene 2018(Artene et al., 2018) | Immunotherapy | high-grade glioma | DC：OS HR，PFS HR；VT:OS HR，PFS HR | DC vaccinations could prolong OS but not PFS. VT could not prolong OS and PFS. |
| Liu 2018(Liu, 2018) | DC vaccine | high-grade glioma | 0.5-，1-，2-，3-，4-year OS RR；0.5-，1-，2-，3-，4-year PFS RR；AE(case) | DC showed better OS and PFS without serious AEs. |
| Cao 2014(Cao et al., 2014) | DC vaccine | high-grade glioma | 1-，1.5-， 2-， 3- and 4-year OS；1-，1.5-， 2-， 3- and 4-year PFS；percentage of CD3^+^ CD8^+^ and CD3^+^CD4^+^ T cells and CD16^+^ lymphocyte；the levels of IFN-γ | DC prolonged OS and PFS and enhanced immune function markedly. |
| Wang 2014(Wang et al., 2014) | DC vaccine | high-grade glioma | 2-year OS OR；AE(case and percentage) | DC prolonged OS and 2-year survival. |
| Zhao 2014(Zhao et al., 2014) | HSV-tk | high-grade glioma | median survival time (MST)；HGG，GBM OS HR；tumor progression OR | Neither the pooled analysis of OS, nor the combined analysis of tumor progress indicates any significant advantage |

Supplementary Table 1. Basic characteristics of studies with existing meta-analyses

Artene, S. A., Turcu-Stiolica, A., Ciurea, M. E., Folcuti, C., Tataranu, L. G., Alexandru, O., et al. (2018). Comparative effect of immunotherapy and standard therapy in patients with high grade glioma: a meta-analysis of published clinical trials. *Scientific Reports* 8, 1–10. doi:10.1038/s41598-018-30296-x.

Cao, J. X., Zhang, X. Y., Liu, J. L., Li, D., Li, J. L., Liu, Y. S., et al. (2014). Clinical efficacy of tumor antigen-pulsed dc treatment for high-grade glioma patients: Evidence from a meta-analysis. *PLoS ONE* 9. doi:10.1371/journal.pone.0107173.

Hanaei, S., Afshari, K., Hirbod-Mobarakeh, A., Mohajer, B., Amir Dastmalchi, D., and Rezaei, N. (2018). Therapeutic efficacy of specific immunotherapy for glioma: a systematic review and meta-analysis. *Reviews in the Neurosciences* 29, 443–461. doi:10.1515/revneuro-2017-0057.

Liu, T. (2018). Efficacy and safety analysis on dendritic cell-based vaccine-treated high-grade glioma patients : a systematic review and meta-analysis. *Oncotargets and Therapy*, 7277–7293.

Wang, X., Zhao, H. Y., Zhang, F. C., Sun, Y., Xiong, Z. Y., and Jiang, X. B. (2014). Dendritic cell-based vaccine for the treatment of malignant glioma: A systematic review. *Cancer Investigation* 32, 451–457. doi:10.3109/07357907.2014.958234.

Zhao, F., Tian, J., An, L., and Yang, K. (2014). Prognostic utility of gene therapy with herpes simplex virus thymidine kinase for patients with high-grade malignant gliomas: A systematic review and meta analysis. *Journal of Neuro-Oncology* 118, 239–246. doi:10.1007/s11060-014-1444-z.
